# Computational identification of potential natural terpenoid inhibitors of MDM2 for breast cancer therapy: molecular docking, molecular dynamics simulation, and ADMET analysis

**DOI:** 10.3389/fchem.2025.1527008

**Published:** 2025-04-16

**Authors:** Eva Azme, Md. Mahmudul Hasan, Md. Liakot Ali, Rashedul Alam, Neamul Hoque, Fabiha Noushin, Mohammed Fazlul Kabir, Ashraful Islam, Tanzina Sharmin Nipun, S. M. Moazzem Hossen, Hea-Jong Chung

**Affiliations:** ^1^ Department of Pharmacy, Faculty of Biological Sciences, University of Chittagong, Chittagong, Bangladesh; ^2^ Department of Biotechnology, Harrisburg University of Science and Technology, Harrisburg, PA, United States; ^3^ Department of Biochemistry and Molecular Biology, Hollings Cancer Center, Medical University of South Carolina, Charleston, SC, United States; ^4^ Honam Regional Center, Korea Basic Science Institute (KBSI), Gwangju, Republic of Korea; ^5^ Department of Bio-Analysis Science, University of Science and Technology, Daejeon, Republic of Korea

**Keywords:** MDM2, breast cancer, ensemble docking, terpenoid, in-silico, MD simulations

## Abstract

**Background:**

Breast cancer (BC) remains a leading cause of cancer-related mortality in women. The oncoprotein MDM2 negatively regulates the tumor suppressor p53, and its overexpression in BC promotes tumor progression and resistance to therapy. Targeting the MDM2-p53 interaction represents a promising therapeutic approach. However, many existing MDM2 inhibitors suffer from poor pharmacokinetics and off-target toxicity, necessitating the discovery of novel, more selective alternatives. This study aims to identify natural terpenoid compounds with potent MDM2 inhibitory potential through computational approaches.

**Methods:**

A library of 398 natural terpenoids was sourced from the NPACT database and filtered based on Lipinski’s Rule of Five. A two-stage docking strategy was applied: 1) rigid protein-flexible ligand docking to screen for high-affinity binders, followed by 2) ensemble docking using multiple MDM2 conformations derived from molecular dynamics (MD) simulations. The top candidates were further evaluated for their pharmacokinetic and toxicity profiles using ADMET analysis. Finally, 150 ns MD simulations and binding free energy (MM-PBSA) calculations were performed to assess the stability and strength of protein-ligand interactions.

**Results:**

Three terpenoid compounds, olean-12-en-3-beta-ol, cabralealactone, and 27-deoxyactein demonstrated strong binding affinities toward MDM2 in ensemble docking studies. ADMET analysis confirmed their favorable pharmacokinetic properties. Further MD simulations indicated that these compounds formed highly stable complexes with MDM2. Notably, 27-deoxyactein exhibited the lowest binding free energy (−154.514 kJ/mol), outperforming the reference inhibitor Nutlin-3a (−133.531 kJ/mol), suggesting superior binding stability and interaction strength.

**Conclusion:**

Our findings highlight 27-deoxyactein as a promising MDM2 inhibitor with strong binding affinity, stability, and a favorable pharmacokinetic profile. This study provides a computational foundation for further experimental validation, supporting the potential of terpenoid-based MDM2 inhibitors in BC therapy.

## Graphical Abstract

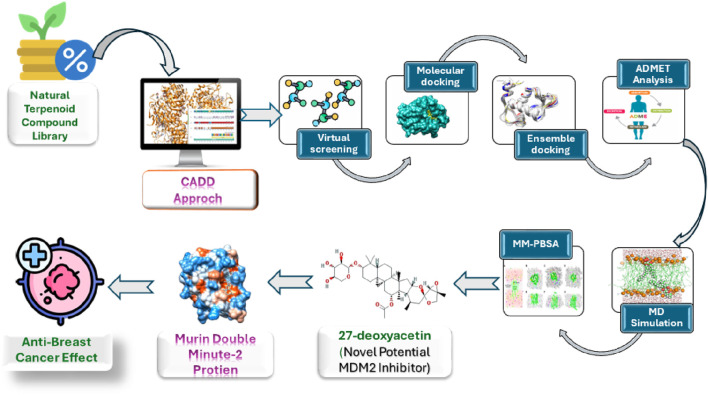

## 1 Introduction

Breast cancer (BC), being the most frequently occurring malignant cancer, is the foremost contributor to cancer-related deaths in women across the world. The incidence of female BC has surpassed that of lung cancer, with around 2.3 million newly reported cases reported worldwide (Sung et al., 2021; Ali et al., 2024c). BC, a diverse set of diseases originating in breast tissue, usually appears as a lump or mass. Primarily, BC develops from the epithelial cells that line the milk duct (Lai and Roy, 2004; Polyak, 2011). Surgery is the main approach for treating BC, with additional options such as chemotherapy, radiation, hormone therapy, targeted therapy, and immunotherapy available. Nonetheless, these treatments may not fully tackle the disease due to negative side effects, the toxicity of chemotherapeutic drugs, resistance or tolerance to therapy, and the progression of BC to metastatic stages (Abdulameed et al., 2023; Shekar et al., 2024). Hence, it is essential to find cost-effective therapeutic methods with minimal adverse effects to enhance the existing treatment options for BC patients. There has been a significant move away from synthetic pharmaceuticals towards natural product-based remedies, and numerous studies have started exploring the anticancer properties of phytochemicals (Ali L. et al., 2024; 2024b). These compounds have demonstrated safety, efficacy, and non-toxicity, making them promising candidates for BC treatment (Svolacchia et al., 2023). Terpenoids, the largest class of natural products, consist of over forty thousand diversified structures mostly derived from plants (Gershenzon and Dudareva, 2007; Rabi and Bishayee, 2009). Comprising five carbon isoprene units, they are further categorized as hemiterpenoids, monoterpenoids, sesquiterpenoids, sesterterpenoids, diterpenoids, triterpenoids as well as polyterpenoids (Huang et al., 2019). Along with, their abundant biological properties, their anti-proliferative, apoptotic, anti-angiogenic, and anti-metastatic properties are well pronounced. The balance of activating and inhibiting certain proteins responsible for cancer initiation and propagation can be modulated by terpenoids (Ghantous et al., 2010; Luo et al., 2019; Ashrafizadeh et al., 2020). Paclitaxel, a terpene-based anticancer drug, is well-known among other terpene-based therapeutics (Howat et al., 2014). Recent findings have shown that terpenoids exhibit dual roles as both chemopreventive and chemotherapeutic agents against BC, displaying multifaceted anticancer properties supported by encouraging outcomes in preclinical research (Bishayee et al., 2011). Several anti-breast cancer agents obtained from terpenoids are japonicone A, inulanolide A, lineariifolianoid A, and hispolon (Qin et al., 2018). However, additional investigations are needed to fully utilize the vast array of compounds with diverse structures to meet the unmet requirements for BC therapy, ensuring both safety and efficacy are sufficiently addressed.

**FIGURE 1:**
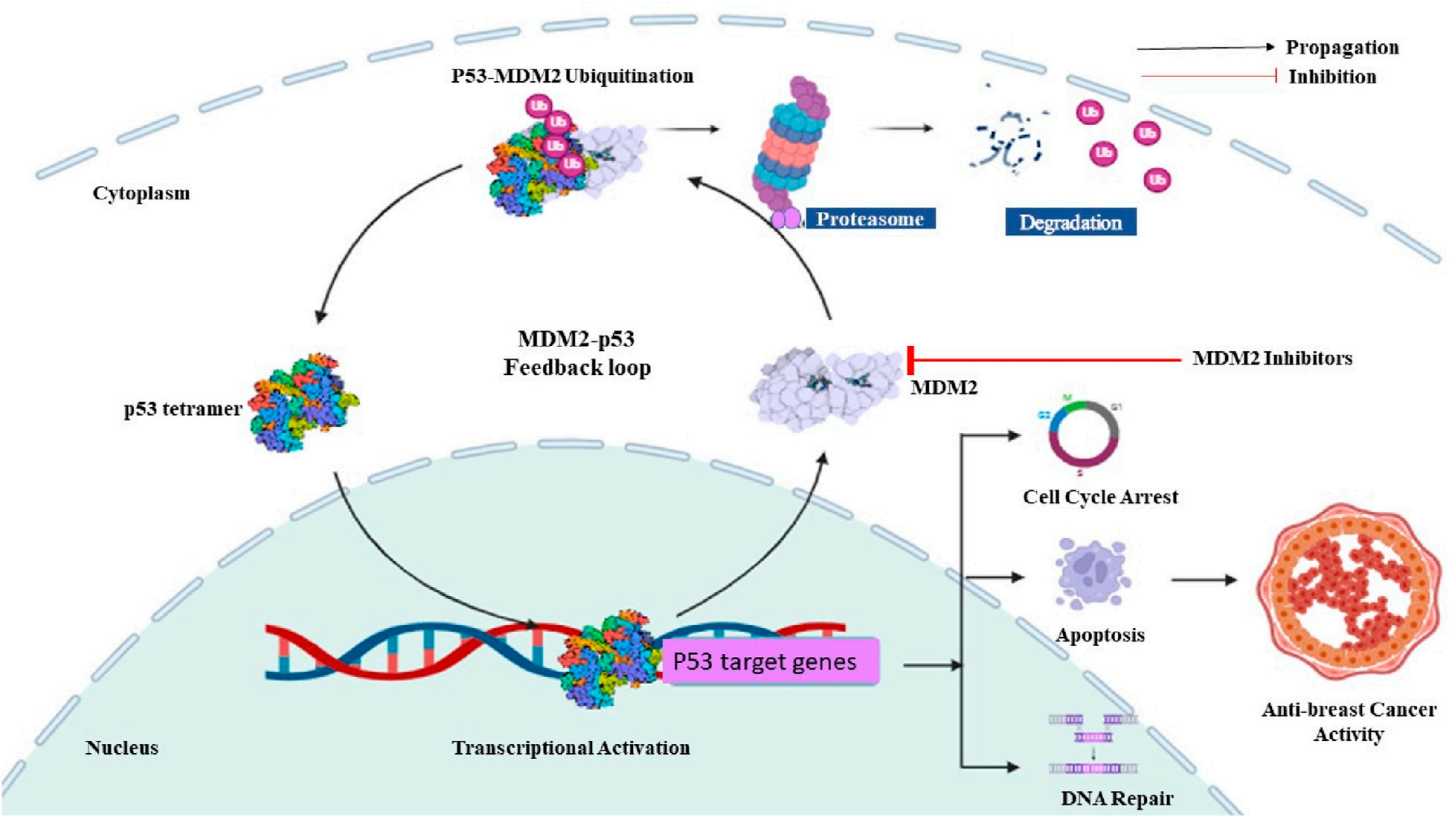
Role of MDM2 in breast cancer. Activation of the p53 protein triggers cell cycle arrest, DNA repair, and apoptosis. However, in breast cancer, heightened levels of MDM2 lead to the ubiquitination of p53, prompting its degradation through proteasomes. To counter this, MDM2 inhibitors can be utilized to impede their interaction with p53, thereby averting its degradation.

Murine double minute 2 (MDM2), an oncoprotein, was discovered by its increased expression in a spontaneously transformed mouse cancer cell line (Kussie et al., 1996; Wade et al., 2010; Popowicz et al., 2011; Wang et al., 2012). MDM2 serves as the primary suppressor of the tumor suppressor p53. In normal cells, p53 restricts MDM2 by attaching to its N-terminal activation domain, and conversely, MDM2 controls p53 by facilitating its degradation via ubiquitin-mediated protein degradation (Fan et al., 2019). This finely tuned auto-regulatory loop regulates low cellular levels of p53 in normal conditions, important for preserving normal cell division and development, thereby emphasizing the intricate interplay between MDM2 and p53 in cellular homeostasis (Wu et al., 2006). However, studies on human cancers have demonstrated that MDM2 is frequently modified and upregulated particularly in BC (Karni-Schmidt et al., 2016). Elevated levels of MDM2 protein have been detected in at least one-third (38%) of cases of human BC. However, the overexpression of MDM2 is particularly prominent in estrogen receptor-positive (ER+) and progesterone receptor-positive (PR+) luminal BC, often occurring alongside wild-type p53 (Yu et al., 2014). MDM2 exerts its tumor-promoting effects in BC through pathways that involve both p53-dependent and p53-independent mechanisms (Haupt et al., 2017). In a p53-dependent manner, MDM2 inhibits the transcriptional activity of p53 by binding to it, thereby preventing its capacity to trigger cell cycle arrest as well as apoptosis (Figure 1). This fosters unregulated cell proliferation characteristic of BC in early tumorigenesis (Nag et al., 2013). Furthermore, MDM2 helps the ubiquitination and subsequent breakdown of p53, further attenuating its tumor-suppressive activities. MDM2 overexpression upsets the delicate balance between MDM2 and p53, promoting oncogenic signaling cascades and bolstering tumor growth (Brooks and Gu, 2006). p53-independent pro-tumor effects of MDM2 in BC encompasses several mechanisms. MDM2 increases the tumor-promoting ERα levels while decreasing those of ERβ, a recently identified tumor suppressor that can decrease BC cell migration potential by upregulating adhesion protein expression (Haupt et al., 2017). MDM2 ubiquitinates and encourages the breakdown of this hormone receptor by forming a complex with ERβ and the coactivator CBP in response to AKT signaling (Sanchez et al., 2013). Furthermore, it was shown that increased MDM2 levels encouraged the ubiquitination and degradation of E-Cadherin, consequently, hastening the aggressiveness of cancer cells (Yang et al., 2006). Moreover, MDM2 overexpression in BC cells was associated with higher expression of matrix metalloprotease 9, a known promoter of invasion and metastasis (Chen et al., 2013). Furthermore, increased MDM2 E3 ligase function can promote therapy resistance via a variety of mechanisms. There is evidence that MDM2 ubiquitinates and degrades SIRT6, a significant tumor suppressor. In human epidermal growth factor receptor 2 positive (HER2+) BC, it was discovered that this contributed to trastuzumab resistance (Thirumurthi et al., 2014). MDM2 overexpression in combination with MDM4 impacted the transcriptional activity of the SMAD family of proteins, preventing TGF-β mediated growth arrest in several BCs that were not responsive to TGF-β. It was a factor in TGF-β resistance (Haupt et al., 2017). These studies highlight the crucial involvement of MDM2 in the development, progression, invasiveness, and resistance to therapy of BC, through both p53-dependent and p53-independent mechanisms, outlining potential therapeutic opportunities for intervention. Several MDM2 inhibitors have been already designed of identified, with the most notable ones being Nutlin-3a, RG7112, MI-63, MI-219, and AMG-232. Despite demonstrating certain levels of effectiveness against the MDM2 protein, none of these inhibitors have received clinical approval thus far due to drawbacks such as limited potency, unfavorable physicochemical characteristics, or inadequate pharmacokinetic properties (Vu et al., 2013; Oyedele et al., 2023). Hence, there is still an expectation for the emergence of more potent MDM2 inhibitors with improved pharmacokinetic properties, holding promise as a valuable addition to the arsenal against BC.

Computer-Aided Drug Design (CADD) employs computational techniques to aid discovery, identification and development new drugs. It accelerates the identification of potential drug molecules, integrates the process, and reduces costs (Hassan Baig et al., 2016). By giving compounds that are most worthy of experimental testing priority, CADD accelerates the search for new drugs as well as enhances the efficacy of drug development process (Song et al., 2009; Huang et al., 2010). CADD study incorporates techniques like molecular docking, ensemble docking, ADMET investigations, and molecular dynamics (MD) simulations (Shah et al., 2024). Molecular docking analysis identifies potential lead candidates based on ligands’ binding scores with proteins. Ensemble docking, a relatively newer computational drug design technique, considers multiple receptor conformations to better capture the structural flexibility of proteins. This method often yields superior results in virtual screening compared to traditional rigid protein-flexible ligand docking approaches (Mohammadi et al., 2022). Once lead compounds are identified, they are screened for their ADMET properties, reducing the need for extensive re-evaluation of pharmacokinetic (PK) attributes and safety profiles such as toxicity and carcinogenicity, thereby minimizing labor. Additionally, MD simulations help assess the stability of protein-ligand interactions (Islam et al., 2024).

In this computational study, a compound library containing 398 natural terpenoid compounds is utilized. Initially, these compounds undergo the Lipinski rule of five (Ro5) filter. Subsequently, a two-step docking process is employed. The first step involves traditional rigid protein-flexible ligand docking, followed by docking of ligands with an ensemble of multiple MDM2 structures to identify potential lead compounds. Following this, ADMET investigations and 150 ns MD simulations are conducted to assess the PK properties as well as safety profiles and stability of protein-ligand interactions for these lead compounds, respectively. The goal of this study is to identify a novel MDM2 inhibitor from the natural terpenoid compounds to treat BC using a comprehensive computational approach.

## 2 Materials and methods

### 2.1 Ligand preparation

A compound library of 398 natural terpenoid compounds (Supplementary Table S1) was collected from the NPACT database (Mangal et al., 2013), which compiles data on naturally occurring plant-derived compounds with proven anti-cancer properties, both *in vitro* and *in vivo*, to supplement other databases. Terpenoid compounds stand out due to their structural diversity, favorable bioavailability, and multimodal anticancer mechanisms, such as apoptosis induction, autophagy modulation, and microtubule stabilization (Kamran et al., 2022). Terpenoids possess polycyclic, rigid frameworks that enable stronger and more stable binding to molecular targets, promoting selective cytotoxicity and minimizing off-target effects (Huang et al., 2012; Yang et al., 2020). Their enhanced lipophilicity compared to alkaloids and polyphenols improves cellular permeability, leading to better absorption, distribution, and overall therapeutic efficacy (Chen et al., 2016). For all the aforementioned reasons, this study aimed to explore novel terpenoid-based compounds as potential MDM2 inhibitors, utilizing their structural advantages to develop more effective and targeted anticancer therapies.

Selected terpenoid compounds underwent screening with the Lipinski Ro5 filter (Lipinski, 2004) to confirm their drug-like characteristics. The canonical SMILES notation of each compound was utilized as input in the SwissADME server (Daina et al., 2017), and compounds that violated no more than one rule of the Lipinski Ro5 were considered to possess drug-like properties. Then, all compounds were obtained from the PubChem database in 3D SDF format. Open Babel GUI software (O’Boyle et al., 2011) was used to convert compounds from 2D SDF format to 3D SDF format whose 3D conformer was not available in the PubChem Database. The minimization of the compounds was accomplished using the mmff94 force field (Tosco et al., 2014). Subsequently, all the compounds were converted into PDBQT format and denoted as ligands.

### 2.2 Protein preparation

The three-dimensional crystal structure of MDM2 was obtained from the RCSB Protein Data Bank (https://www.rcsb.org/), and its PDB ID is 4HG7 (Resolution: 1.60 Å). Using Biovia Discovery Studio (Studio, 2008), undesirable atoms, and water molecules were cleaned up. After that, a universal forcefield GROMOS96 (Lemkul et al., 2010) was applied to minimize the protein in the SPDB viewer (Guex and Peitsch, 1997) to evaluate the protein’s missing h-bond, side-chain abnormalities, and incorrect bonds. Finally, the protein was ready for additional computational procedures such as molecular docking.

### 2.3 Molecular docking

Molecular docking is an essential technique in CADD and structural molecular biology (Sany et al., 2025). The purpose of ligand-protein docking is to apprehend the predominant binding modes between a ligand and a drug-target protein (Morris and Lim-Wilby, 2008; Ali Md. L. et al., 2024). The compounds varied in their interactions and binding affinities with the target protein. Using the Broyden–Fletcher–Goldfarb–Shanno algorithm, Autodock Vina (Eberhardt et al., 2021), a built-in docking tool in PyRx software (Dallakyan and Olson, 2015), was used for molecular docking. All of the ligands and the protein’s 3D structure were imported into PyRx software and changed to PDBQT format. As a reference molecule, the co-crystallized ligand Nutlin-3a (a recognized inhibitor of MDM2) was also included in the compound library. Nutlin-3a, being a standard inhibitor of MDM2, binds with crucial residues within the MDM2 protein referred to as the active site. A grid box was positioned to cover the complete extent of this active site. Before docking ligands into the active site of the protein, the docking procedure is validated by redocking the co-crystallized ligand, Nutlin-3a into the MDM2 protein’s binding pocket. Then, the co-crystallized ligand’s lowest energy pose, produced by Autodock Vina, was superimposed over the experimental binding pose identified by X-ray crystallography. Subsequently, the root mean square deviation was calculated and the RMSD value had to be less than 2 Å (Acharya et al., 2019). After the docking validation procedure, the grid box of dimensions (25 × 25 × 25) Å, including the grid box center, was set to “X = − 23.273” “Y = − 8.1073”, and “Z = − 13.6129” for the MDM2 protein. Based on their highest negative scores, which indicated the highest binding affinities, the compound’s docking results were computed and ranked (Hossain et al., 2023). A collection of nine unique bound conformations, ascertained by the binding affinity of each ligand. For more inquiry, only the conformational states exhibiting the lowest binding energy for the ligand molecules were chosen. From the docking procedures involving the crystal structure of MDM2 protein, compounds were identified with binding energies lower than that of the standard inhibitor, Nutlin-3a. Subsequently, the best receptor-ligand interactions were visualized and characterized using Discovery Studio.

### 2.4 Ensemble docking

When employing MD simulation to generate an “ensemble” of drug target conformations for docking candidate ligands in computational structure-based drug discovery, this process is known as ensemble docking (Amaro et al., 2018). Using an ensemble of different protein structures is one way to include protein flexibility in molecular docking. It is computationally impractical to sequentially dock each ligand into a large number of protein structures, which hampers large-scale database screening (Huang and Zou, 2007). That’s why the term ’ensemble docking’ was introduced into our work procedure.

After the bound ligand was eliminated from the structure of the crystallographic protein, the protein with a well-equilibrated and solvated system underwent a 250 ns MD simulation. All parameters were the same as “Molecular dynamics simulations” section. Approximately 25,001 post-MD study trajectories were grouped into 15 conformations using a 1.25 Å root mean square deviation cutoff using the *gmx cluster* module (Supplementary Table S3). Top three conformations were taken for ensemble docking as these three conformers represent 99% of the total structure population. The ensemble docking was carried out using the Autodock Vina program (Trott and Olson, 2010) which included multiple protein conformations and multiple ligands. The 3 conformers, obtained by clustering MD simulation trajectory data, were docked with the top compounds obtained from the previous rigid protein-flexible ligand docking procedure. In this case, active site-specific docking procedures were utilized and a grid box with a dimension of (25 × 25 × 25) Å was placed in a way that it covered critical active site residues of this MDM2 protein such as Leu-54, Leu-57, Gly-58, Ile-61, Met-62, Val-93, His-96, and Ile-99 (Chukwuemeka et al., 2022; Oyedele et al., 2023). Following that, the top three compounds were identified by considering their lowest average binding energies across all three protein conformers.

### 2.5 In-silico ADMET investigations

The efficacy and safety of a drug candidate are largely dependent on the absorption, distribution, metabolism, and excretion (ADME) parameters and several toxicities (T). Hence the evaluation of ADMET properties is crucial to minimize the failures in drug discovery at the R&D phase (Dong et al., 2018). It's been noted that ligands demonstrating antagonistic reactions to target proteins may not necessarily be as effective as drugs. Therefore, in CADD, the assessment of PK properties plays a vital role in determining the applicability of drug candidates in biological systems. Furthermore, PK properties help evaluate the efficacy and integrity of compounds during the initial phases of CADD. The analysis of PK properties for our chosen compounds was conducted using the SwissADME server (Daina et al., 2017) and admetSAR 1.0 server (Cheng et al., 2012). Furthermore, evaluating the potential adverse effects of chemical compounds is a critical aspect of drug development prior to clinical trials. Therefore, toxicity assessment is an indispensable component of the drug design process. In this study, the SMILES notation of the compounds was inputted into the ProTox-II web server (Banerjee et al., 2018) to predict the *in silico* toxicity properties of the selected compounds.

### 2.6 Molecular dynamics simulations

MD simulation is the simulation of moving system particles inserted into macromolecules to determine variations from the relative positions of atoms in proteins over time (Karplus and Petsko, 1990). The highly reliable method of MD simulation is utilized to visualize and assess the stability of macromolecules such as proteins and nucleic acids within the constantly moving human body (Hospital et al., 2015). Apo protein, protein-ligand complexes of selected top three compounds, and reference molecule, Nutlin-3a were subjected to MD simulation utilizing Gromacs 2023.3 software (Abraham et al., 2015) to determine their stability. The topology for proteins was generated using the CHARMM27 all-atom force field (Bjelkmar et al., 2010), while the SwissParam server (Bugnon et al., 2023) was employed to generate the topology for ligands. Following topology generation for both ligands and proteins, the complex was solvated using the TIP3P water model and neutralized by adding four Cl^−^ions. Subsequently, equilibration was performed using both canonical NVT and isobaric-isothermal NPT ensembles. The final MD simulation run for the protein-ligand complexes lasted 150 ns under the NPT ensemble, with a temperature of 300 K and a pressure of 1 bar, as previously described (Vegad et al., 2023). The “*trjconv*” module was used to centralize and compact the protein in the resultant MD run trajectory files. Root mean square deviation (RMSD), root mean square fluctuation (RMSF), solvent accessible surface area (SASA), and radius of gyration (RG) of protein as well number of hydrogen bonds (H-bond) were formed between each ligand and proteins, were calculated and plotted to comprehend the molecular-level dynamics alterations of the protein-ligand complexes. Subsequently, principal component analysis (PCA) is performed with the help of *gmx covar* and *gmx anaeig* tools. Using the *gmx covar* tool, the covariance matrix for the apo protein and each complex was created, yielding diagonal eigenvectors that show associated motions within the protein. The value of each eigenvector is represented by the corresponding eigenvalues, which provide information about the atomic contributions to the motion of the protein-ligand complex system. Using the *gmx anaeig* tool, 2D projections of the trajectory were created for visualization by superimposing the first two principal components (Kar et al., 2023). The *gmx sham* package was implemented to calculate the two-dimensional illustrations of the free energy landscapes (FEL) (Altis et al., 2008).

### 2.7 MM-PBSA binding free energy calculation

An open-source program called g_mmpbsa can read the trajectories produced by GROMACS and use the MM-PBSA method to estimate the binding free energy of the protein-ligand complex (Kumari et al., 2014). The binding free energy of the protein-ligand complex can be calculated using Equation 1, as described below:

$$\Delta G_{\text{bind}}=\Delta E_{\text{MM}}+\Delta \;\Delta G_{\text{sol}}\;–\;T\Delta S$$ (1)

Where, ΔG_bind_ is the notation for the binding free energy, ΔS represents the change in entropy, T is the absolute temperature, Δ ΔGsol is the distinction in solvation-free energy, and ΔE_MM_ is the variation in intramolecular energy in a vacuum. By using the “g_mmpbsa” tool, the binding free energies of the protein-ligand complexes were determined at 10 ps intervals for the last 10 ns of MD simulations (total 1000 frames).

## 3 Results

### 3.1 Docking validation

**FIGURE 2:**
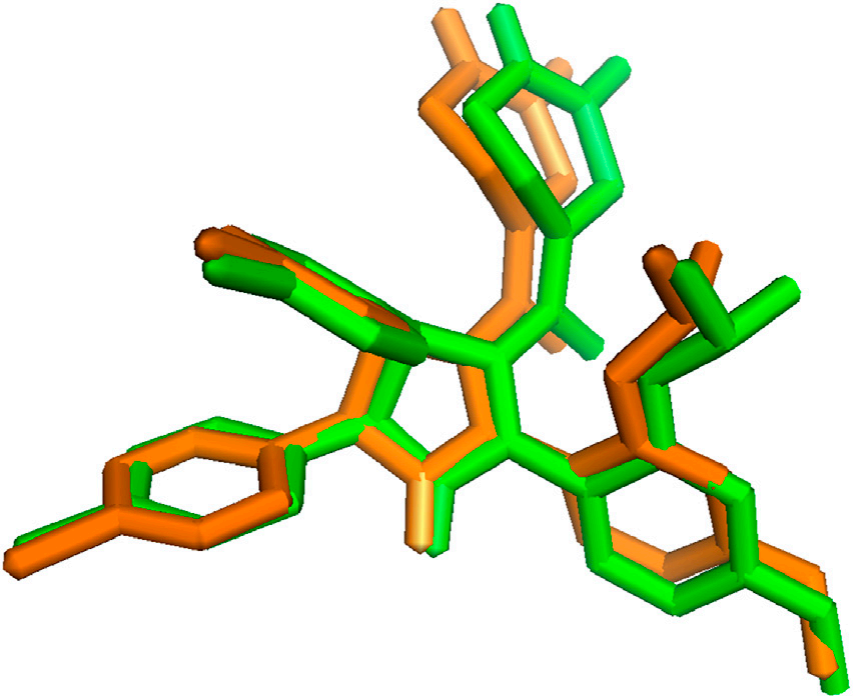
Superimposition of co-crystallized ligand before (green) and after (orange) docking (RMSD = 0.243 Å).

Molecular docking procedure authentication is a critical step in the virtual screening of drugs used to predict the validity and precision of the docking technique (Mukherjee et al., 2010). Hence, to verify the docking process, the reference ligand’s lowest energy pose, which was acquired via Autodock Vina, was compared to an empirically determined binding pose using X-ray crystallography. The maximum dependability of the docking technique is indicated by an RMSD of 0.243 Å between the docked position and the experimental pose, which is within a range of 2 Å (Acharya et al., 2019). By superimposing the two poses, as depicted in Figure 2, the resemblance between the two conformations can be observed.

### 3.2 Molecular docking

Molecular docking, a computational method utilized in medicinal chemistry and drug discovery, predicts the arrangement and binding strength of a small molecule (ligand) with a target protein or biomolecule (receptor). This method plays a pivotal role in screening large compound libraries, prioritizing potential lead compounds, and optimizing their structures for enhanced binding affinity which aids in the rational design of novel therapeutic agents by providing insights into the molecular mechanisms of ligand-protein interactions (Agarwal and Mehrotra, 2016).

**FIGURE 3:**
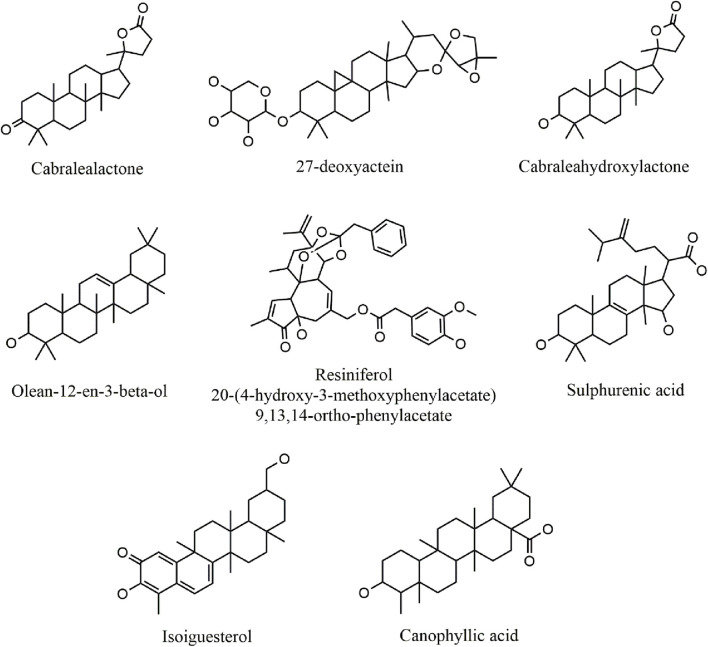
2D structure of selected top-docked terpenoid compounds.

**FIGURE 4:**
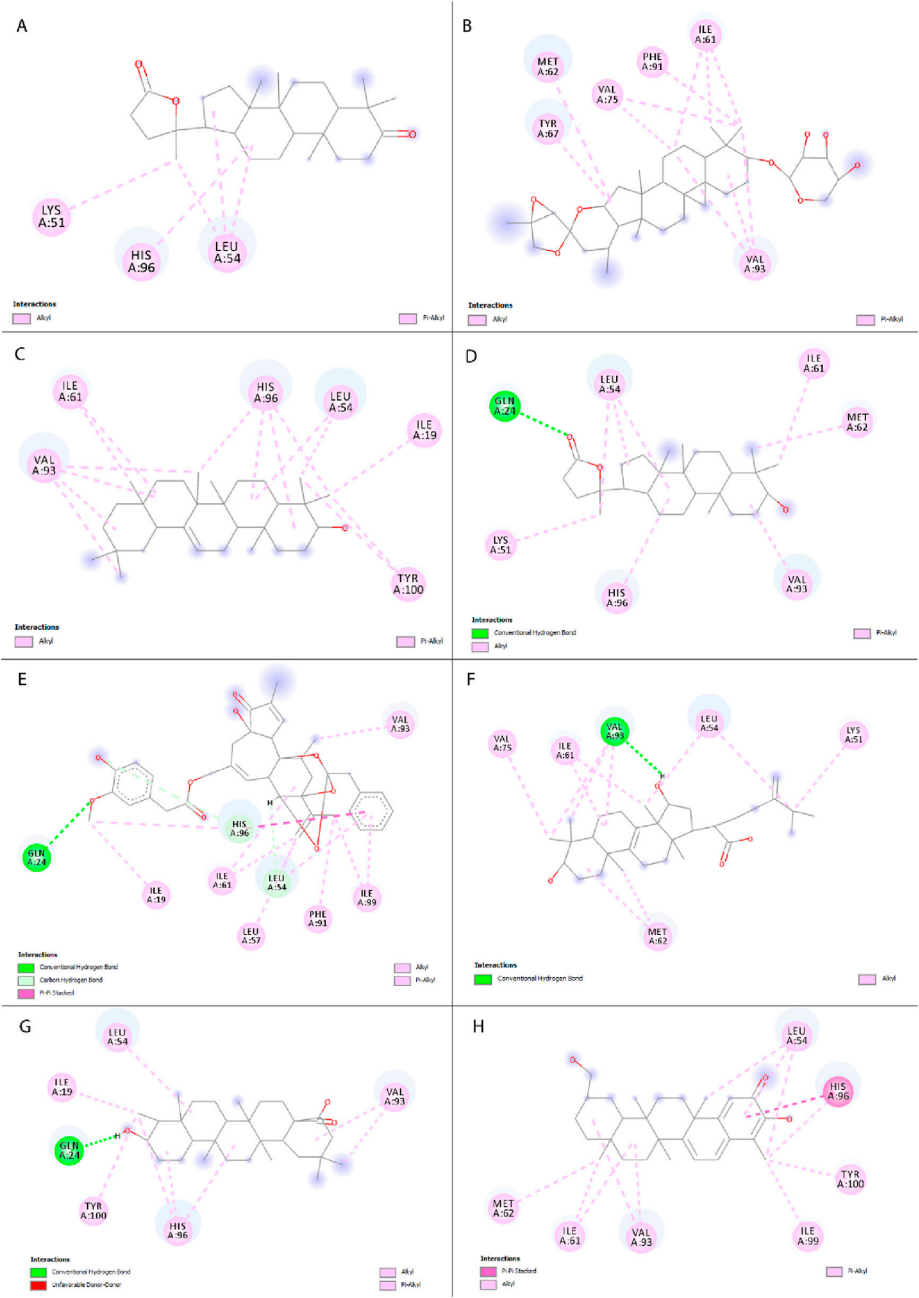
Two-dimensional representation of interactions between selected compounds with MDM2 protein. **(A)** Cabralealactone, **(B)** 27-deoxyactein, **(C)** Olean-12-en-3-beta-ol, **(D)** Cabraleahydroxylactone, **(E)** Resiniferol 20-(4-hydroxy-3-methoxyphenylacetate) 9,13,14-ortho-phenylacetate, **(F)** Sulphurenic acid, **(G)** Canophyllic acid, and **(H)** Isoiguesterol.

A total of 330 compounds were selected for molecular docking after filtering by Lipinski Ro5 (Supplementary Table S1). These compounds did not violate more than one rule of Lipinski Ro5 and are considered to have drug-likeness attributes. These compounds and the reference molecule, Nutlin-3a were docked into the active site of the MDM2 protein. A total of 19 terpenoid compounds showed more favorable binding affinity than standard inhibitor, Nutlin-3a and were subjected to further screening process (Supplementary Table S2). Among these compounds, 4 compounds, namely, lupeol (PubChem Id: 259846) (Muhammad et al., 2020), alpha-amyrin (PubChem Id: 73170) (Victory et al., 2018), celastrol (PubChem ID:122724) (Aziz et al., 2021), balsaminoside A (PubChem ID: 44555454) (Gatti et al., 2011), were already investigated regarding MDM2. Moreover, seven more compounds were also found to have toxicity issues (carcinogenic or mutagenic) during our initial toxicity investigations. Following the exclusion of four compounds that were previously studied and seven compounds identified as toxic, 8 compounds were chosen as potential inhibitors of MDM2 and underwent additional evaluation. 2D structures and two-dimensional protein-ligand interactions of these eight compounds are depicted in Figure 3 and Figure 4, respectively. The binding energies and analysis of protein-ligand interactions for these eight docked compounds and the standard inhibitor, Nutlin-3a, are presented in Table 1.

**TABLE 1:** Molecular docking results of selected top docked terpenoid compounds and reference molecule, Nutlin-3a with MDM2 protein.

SN	Compounds	PubChem ID	Binding energy (kcal/mol)	Interacting residues	
				Hydrogen bonds	Hydrophobic interactions
1	Cabralealactone	44421647	−8.6	None	Leu-54, Lys-51, His-96
2	27-deoxyactein	6537501	−8.6	None	Val-75, Val-93, Ile-61, Phe-91, Met-62
3	Olean-12-en-3-beta-ol	6708529	−8.5	None	Val-93, Leu-54, Ile-19, Ile-61, His-96, Tyr-100
4	Cabraleahydroxylactone	44421648	−8.4	Gln-24	Val-93, Leu-54, Ile-61, Met-62, Lys-51, His-96
5	Resiniferol 20-(4-hydroxy-3-methoxyphenylacetate) 9,13,14-ortho-phenylacetate	53776296	−8.4	Gln-24, Leu-54 Lys-51	Val-93, Ile-19, Ile-99, Leu-54, Leu-57, Ile-61, Phe-91, His-96
6	Sulphurenic acid	5321559	−8.4	Val-93	Ile-61, Val-93, Met-62, Leu-54, Val-75, Lys-51
7	Canophyllic acid	596679	−8.4	Gln-24	Leu-54, Val-93, Ile-19, His-96, Tyr-100
8	Isoiguesterol	10477355	−8.3	None	Leu-54, His-96, Val-93, Ile-61, Met-62, Ile-99, Tyr-100
9	Nutlin-3a (Standard inhibitor)		−8.2	None	Tyr-100, His-96, Val-93, Leu-54, Leu-57, Ile-61, Ile-99

*Active site residues of MDM2 protein are marked as bold.

Previously, Chukwuemeka et al. identified three key sub-pockets within the hydrophobic cleft of the MDM2 protein, crucial for its binding with p53. The first sub-pocket is composed of amino acids Ile-61, Met-62, and Val-93. The second region includes three amino acid residues Leu-57, Gly-58, and Ile-99. Lastly, the third sub-pocket comprises Leu-54 and His-96. It's important to note that the attachment of small molecules or ligands to any of these three sub-pockets would hampers the dimerization process between MDM2 and p53 (Chukwuemeka et al., 2022).

Standard inhibitor, Nutlin-3a showed a binding energy of – 8.2 kcal/mol to the MDM2, which served as a benchmark. It stabilized its protein-ligand complex by forming twelve hydrophobic contacts with seven distinct amino acid residues of MDM2 protein, including Tyr-100, His-96, Val-93, Leu-54, Leu-57, Ile-61, Ile-99. In our investigation, the eight top-docked compounds exhibited a binding pattern resembling that of the standard inhibitor. Cabralealactone (– 8.6 kcal/mol) and 27-deoxyactein (– 8.6 kcal/mol) showed the highest binding affinity toward MDM2 protein, followed by olean-12-en-3-beta-ol (– 8.5 kcal/mol) and cabraleahydroxylactone (– 8.4 kcal/mol).

Cabralealactone (– 8.6 kcal/mol), a natural compound found in *Cleome brachycarpa* (Malik et al., 2018), exhibited five hydrophobic interactions with the MDM2 protein, all of which were hydrophobic. Specifically, this compound formed three alkyl interactions with Leu-54, as well as one alkyl interaction with Lys-51 and one pi-alkyl interaction with His-96. Another top compound, 27-deoxyactein (– 8.6 kcal/mol), a compound extracted from *Cimicifuga racemose* (Suh et al., 2018), exhibited superior hydrophobic interactions with the protein compared to the previous compound. It formed eleven hydrophobic interactions with the protein’s active site. Specifically, it engaged in three alkyl interactions each with Ile-61 and Val-93. Additionally, two alkyl interactions with Val-75 and one alkyl interaction with Met-62 were observed. Furthermore, two pi-alkyl interactions were noted, one with Phe-91 and another with Tyr-67. Olean-12-en-3-beta-ol (− 8.5 kcal/mol), a compound isolated from *Dianthus basuticus* (Nafiu and Ashafa, 2017), established fifteen hydrophobic interactions with the MDM2 protein, five of which were hydrophobic (four interactions with His-96 and one with Tyr-100). Additionally, it formed one alkyl bond each with Tyr-100 and Ile-19. Furthermore, this compound engaged in multiple interactions with Leu-54 (two alkyl interactions), Ile-61 (two alkyl interactions), and Val-93 (four alkyl interactions). Cabraleahydroxylactone (– 8.4 kcal/mol), a compound isolated from *Aglaia exima* (Loong et al., 2010), established one hydrogen bond with Gln-24 and a pi-alkyl interaction with His-96. It also engaged in three alkyl interactions with Leu-54, as well as one interaction each with Lys-51, Ile-61, Met-62, and Val-93. Resiniferol 20-(4-hydroxy-3-methoxyphenylacetate) 9,13,14-ortho-phenylacetate (a derivative of resiniferatoxin), sulphurenic acid (a triterpenoid compound from *Antrodia camphorata*) (Lin et al., 2019), and canophyllic acid (a bioactive molecule from *Calophyllum inophyllum*) (Prasad et al., 2012) exhibited the same binding energy of – 8.4 kcal/mol. However, while resiniferol 20-(4-hydroxy-3-methoxyphenylacetate) 9,13,14-ortho-phenylacetate formed three hydrogen bonds (one each with Gln-24, Leu-54, and Lys-51), sulphurenic acid, and canophyllic acid only formed one hydrogen bond each. These three compounds showed several hydrophobic interactions with active site residues, especially with Leu-54, Ile-61, Val-93, and His-96. Finally, isoiguesterol, a phytosterol isolated from *Salacia kraussii* (Figueiredo et al., 1998), exhibited a binding energy of – 8.3 kcal/mol and engaged in numerous interactions with the MDM2 protein. All of these interactions were hydrophobic and involved residues such as Leu-54, His-96, Val-93, Ile-61, Met-62, Ile-99, and Tyr-100.

Here, every selected top compound exhibited hydrophobic bond interactions with active site residues such as Leu-54, Leu-57, Gly-58, Ile-61, Met-62, Val-93, His-96, and Ile-99, indicating a potential disruption of MDM2-p53 dimerization and suggesting a viable therapeutic strategy for altering the MDM2-p53 interaction in BC treatment.

### 3.3 Ensemble docking

In the realm of molecular docking, ensemble docking is a computer-aided method that takes into account several protein receptor conformations or structures to predict the binding mechanism and affinity of ligands to a target protein (Amaro et al., 2018). In contrast to conventional molecular docking, which usually utilizes a single, static protein structure, ensemble docking acknowledges and takes into consideration the intrinsic flexibility and dynamic characteristics of biomolecules (Huang and Zou, 2007). Numerous investigations have indicated that protein structures generated from MD simulations yield superior docking results than protein structures docked to the crystal structure (Osguthorpe et al., 2012).

**FIGURE 5:**
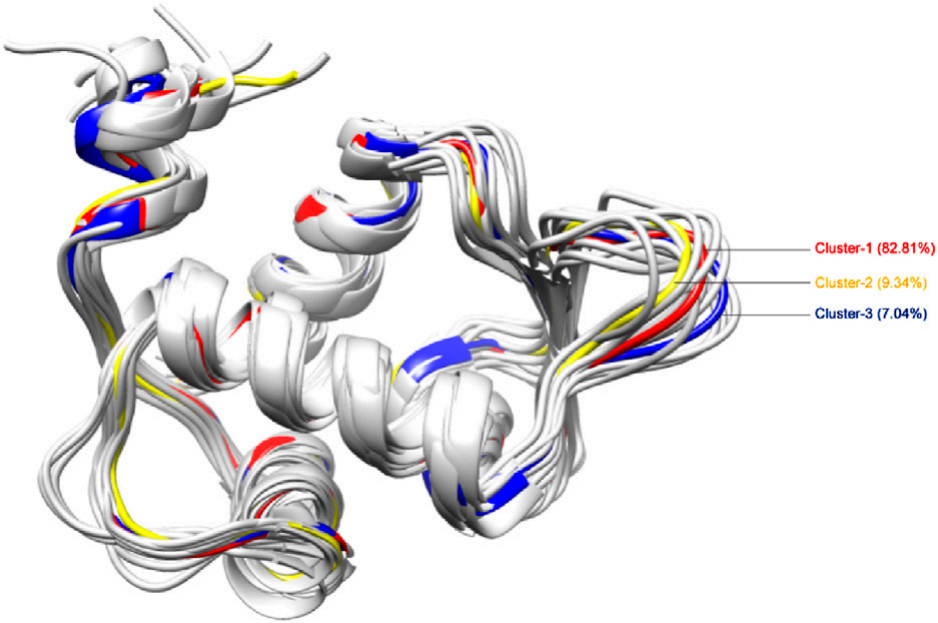
3D representation of structural conformations of MDM2 protein generated by clustering of MD simulation trajectory data. Cluster-1, the largest, constituted 82.81% of all conformations; Cluster-2 accounted for 9.34%; and Cluster-3 comprised 7.04%. Cluster-1, cluster-2, and cluster-3 are indicated in red, yellow, and blue colors respectively.

We employed 250 ns MD trajectories of the unbound MDM2 protein to generate an ensemble of protein structures, capturing the varied conformations of binding site residues. Employing a clustering cutoff of 1.25 Å, we identified 15 clusters from the MDM2 protein simulation (Supplementary Table S3). Figure 5 presents the 3D visualization of the central structures from all these clusters. However, only the top three clusters (Cluster-1, Cluster-2, and Cluster-3) were chosen, which represented the majority of the simulation trajectory. Cluster-1, comprising the highest number of confirmations, accounted for 82.81% of the total members. Moreover, Cluster-2 and Cluster-3 represented 9.34% and 7.04% of the total conformations respectively. Together, these three clusters encompassed over 99% of the conformations, encompassing most of the principal conformations of the protein’s structural changes. This justified the selection of the top three representative structures using clustering. Further analysis focused on the middle structure from each of these three clusters to capture the diversity in the conformation of the binding site.

Table 2 highlights the binding energies of docked compounds with representative conformations of all three clusters, with Nutlin-3a serving as the standard inhibitor to provide a point of comparison. Remarkably, as compared to Nutlin-3a, all eight compounds exhibit greater binding affinities, except for sulphurenic acid.

**TABLE 2:** List of Compounds included in ensemble docking along with their binding energies.

SN	Compound name	PubChem ID	Binding energy (kcal/mol)			Average binding energy (kcal/mol)
			Cluster-1	Cluster-2	Cluster-3	
1	Olean-12-en-3-beta-ol	6708529	−7.9	−8.2	−8.3	−8.13
2	27-deoxyactein	6537501	−8.3	−7.9	−7.6	−7.93
3	Cabralealactone	44421647	−8.5	−7.8	−7.5	−7.93
4	Canophyllic acid	596679	−8.7	−8	−6.6	−7.77
5	Resiniferol 20-(4-hydroxy-3-methoxyphenylacetate) 9,13,14-ortho-phenylacetate	53776296	−8.3	−7.6	−7.2	−7.70
6	Cabraleahydroxylactone	44421648	−8	−7.5	−7.1	−7.60
7	Isoiguesterol	10477355	−7.6	−7.5	−6.8	−7.30
8	Nutlin-3a (Standard inhibitor)	11433190	−7.3	−6.8	−6.1	−6.73
9	Sulphurenic acid	5321559	−6.8	−6.3	−5.9	−6.33

Among these compounds, olean-12-en-3-beta-ol was the compound with the highest binding affinity in the ensemble docking data, with an average docking score of – 8.13 kcal/mol across all three clusters, despite not showing particularly high binding affinities within Cluster-1. Moreover, 27-deoxyactein and cabralealactone stood out for their considerable affinity across all three clusters, as indicated by their optimal average binding energy of – 7.93 kcal/mol. Strong interactions are suggested by the constant or marginally enhanced binding energies for 27-deoxyactein, olean-12-en-3-beta-ol, and cabralealactone across clusters. In contrast, canophyllic acid showed a significant drop in binding energy in Cluster-3, indicating a weaker interaction at that particular binding site. The rest of the compounds exhibited comparatively higher average binding energies, suggesting a lower overall binding affinity.

These results suggest that olean-12-en-3-beta-ol, 27-deoxyactein, and cabralealactone exhibited the strongest binding affinities towards the principal conformations of the MDM2 protein. Therefore, they hold potential as promising candidates for further exploration and lead optimization in drug discovery endeavors.

### 3.4 ADMET investigations

ADMET analysis is an essential step of drug discovery that determines the potential path of how our body will eventually treat the drug material and if this drug is safe to use or not. It is very crucial to determine the pharmacokinetics as well as the drug-likeliness profile of a chemical compound at the very early stage of drug discovery because it has been one of the leading causes of failure in clinical trials (Dong et al., 2018). Moreover, toxicological features of selected compounds including, hepatotoxicity, AEMS mutagenicity, carcinogenicity, and cytotoxicity were analyzed via Protox II webserver. Table 3 shows all the parameters related to PK characteristics as well as the toxicity profiles of our selected compounds and standard inhibitor, nutlin-3a.

**TABLE 3:** ADMET profiling of selected terpenoid compounds as potential inhibitors of MDM2.

Properties	Olean-12-en-3-beta-ol	27-Deoxyacetein	Cabralealactone	Nutlin-3a (standard inhibitor)
Molecular weight	426.72 g/mol	602.80 g/mol	414.62 g/mol	581.49 g/mol
Hydrogen bond acceptors	1	8	3	5
Hydrogen bond donors	1	3	0	1
Log P value	7.18	4.1	5.61	4.53
Rotatable Bond	1	2	1	8
TPSA	20.23 Å^2^	110.14 Å^2^	43.37 Å^2^	83.47 Å^2^
Lipinski violations	1	1	1	2
Verber Rule Violations	0	0	0	0
Pains Alert	0 alert	0 alert	0 alert	0 alert
Bioavailability Score	0.55	0.55	0.55	0.17
Blood-Brain Barrier	No	No	No	No
GI absorption	Low	High	High	High
Synthetic accessibility	6.04	8.73	5.1	5.16
CYP3A4 inhibitor	Non-inhibitor	Non-inhibitor	Non-inhibitor	Inhibitor
CYP2D6 inhibitor	Non-inhibitor	Non-inhibitor	Non-inhibitor	Non-Inhibitor
hERG inhibition	Weak inhibitor	Weak inhibitor	Weak inhibitor	Weak inhibitor
AMES Toxicity	Non-AMES Toxicity	Non-AMES Toxicity	Non-AMES Toxicity	Non-AMES Toxicity
Carcinogenicity	Non-carcinogens	Non-carcinogens	Non-carcinogens	Non-carcinogens
Hepatotoxicity	Non-hepatotoxic	Non-hepatotoxic	Non-hepatotoxic	Non-hepatotoxic
Cytotoxicity	Non-cytotoxic	Non-cytotoxic	Non-cytotoxic	Non-cytotoxic
Predicted LD50	2.0842 mol/kg	3.3088 mol/kg	2.4518 mol/kg	2.5907 mol/kg

Analyzing the drug-likeness profile of a chemical compound is equally indispensable because it estimates whether the compound holds suitable properties for being employed as an orally active drug (Lipinski et al., 1997). To stick to the drug-likeness profile, a chemical compound must follow the Lipinski Ro5 without violating more than one rule. This rule implies that a good drug candidate must follow these five laws including- 1) holding molecular mass less than 500 Da 2) Log P should not be less than 53) Total polar surface area (TPSA) should not be greater than 140 Å (4) Hydrogen bond doner (HBDs) should not be less than 5 and 5) Hydrogen bond acceptors (HBAs) should not be less than 10 (Anza et al., 2021). On the other hand, Verber’s rule is another important parameter to determine oral absorption of a drug molecule which states that a chemical compound should possess 10 or less than 10 rotatable bonds and should hold a surface area not more than 140 Å to exert good oral absorption (Hou et al., 2007). Here the number of rotatable bonds of all the compounds is less than 10 which makes them conformationally stable (Abdelrheem et al., 2021). Based on the obtained results, selected compounds were perfectly eligible to be utilized as drug molecules as all of them adhered to Lipinski and Verber’s rules. However, Nutlin-3a presented two Lipinski violations, whereas the selected terpenoids had only one, indicating a slight advantage in their drug-likeness profile.

A comparative analysis between the three selected terpenoid compounds and the reference molecule Nutlin-3a reveals key differences and similarities in their ADMET profiles. Nutlin-3a, a well-established MDM2 inhibitor, demonstrated high GI absorption similar to 27-Deoxyacetein and Cabralealactone, whereas Olean-12-en-3-beta-ol exhibited low GI absorption, which may impact its bioavailability. Furthermore, our evaluation revealed that none of the top selected compounds could cross the blood-brain barrier (BBB), indicating a reduced risk of central nervous system adverse effects. Moreover, all the chosen compounds exhibited satisfactory bioavailability scores, with values reaching up to 55%. Pan-Assay Interference Compounds (PAINS) are substances responsible for producing false positive results during the initial virtual screening process of the drug discovery stage which is often misleading. However, all of the compounds here had been reported to show zero PAINS alert. The three respective compounds we selected had not demonstrated any inhibition or interference with enzymes CYP3A4 and CYP2D6; two vital enzymes that account for the metabolism of 30% of prescribed drugs (Zanger and Schwab, 2013). In contrast, Nutlin-3A was identified as a CYP3A4 inhibitor, suggesting a higher potential for drug-drug interactions compared to the selected terpenoids. Therefore, the selected compounds were expected to undergo metabolism easily in the body without generating any toxic metabolites.

It has been established that toxicity is one of the key concerns in the drug discovery and development phase (Xu et al., 2012). Therefore, investigating the toxicological profile of a drug at the preclinical stage is extremely important to ensure safe and efficacious drug delivery. Carcinogenicity, hepatotoxicity, acute toxicity, and AMES mutagenicity are four fundamental types of toxicities that make a chemical compound unsuitable to be utilized as a drug molecule if any of them are present in that compound. In our investigation, three selected terpenoid compounds were thoroughly free from the risk of creating hepatotoxicity, carcinogenicity, cytotoxicity, and AEMS mutagenicity like the reference molecule, Nutlin-3a. Many of the marketed drugs are being withdrawn due to causing severe adverse hepatic side effects (Low et al., 2011). Fortunately, none of our experimental compounds have shown to cause hepatotoxicity.

A major constraint in the path of drug development is the inhibition of Human ether-a-go-go-related-gene (hERG) which causes massive cardiotoxicity (Saxena et al., 2016). Three of our selected compounds had shown a very weak extent of hERG inhibition activity which indicated that their possibility to exert cardiotoxicity was very negligible though further detailed investigation is required. Another important parameter to determine acute toxicity is the median lethal dose, LD50 value (dose of an experimental compound that kills 50% of the tested animal population at a specific period) (Lei et al., 2016). Based on our assessment, all the compounds have demonstrated a moderate level of LD50 values, indicating their safety. However, Nutlin-3a had a lower predicted LD50 than 27-Deoxyacetein, suggesting the latter may have a higher safety margin.

Thus, while Nutlin-3a remains a potent reference inhibitor, the selected terpenoids exhibit comparable or even superior pharmacokinetic and safety profiles, making them promising candidates for further drug development.

So, ADMET analysis revealed that all three selected compounds displayed favorable PK properties and safety profiles, indicating their potential as promising drug candidates.

### 3.5 Molecular dynamics simulations

**FIGURE 6:**
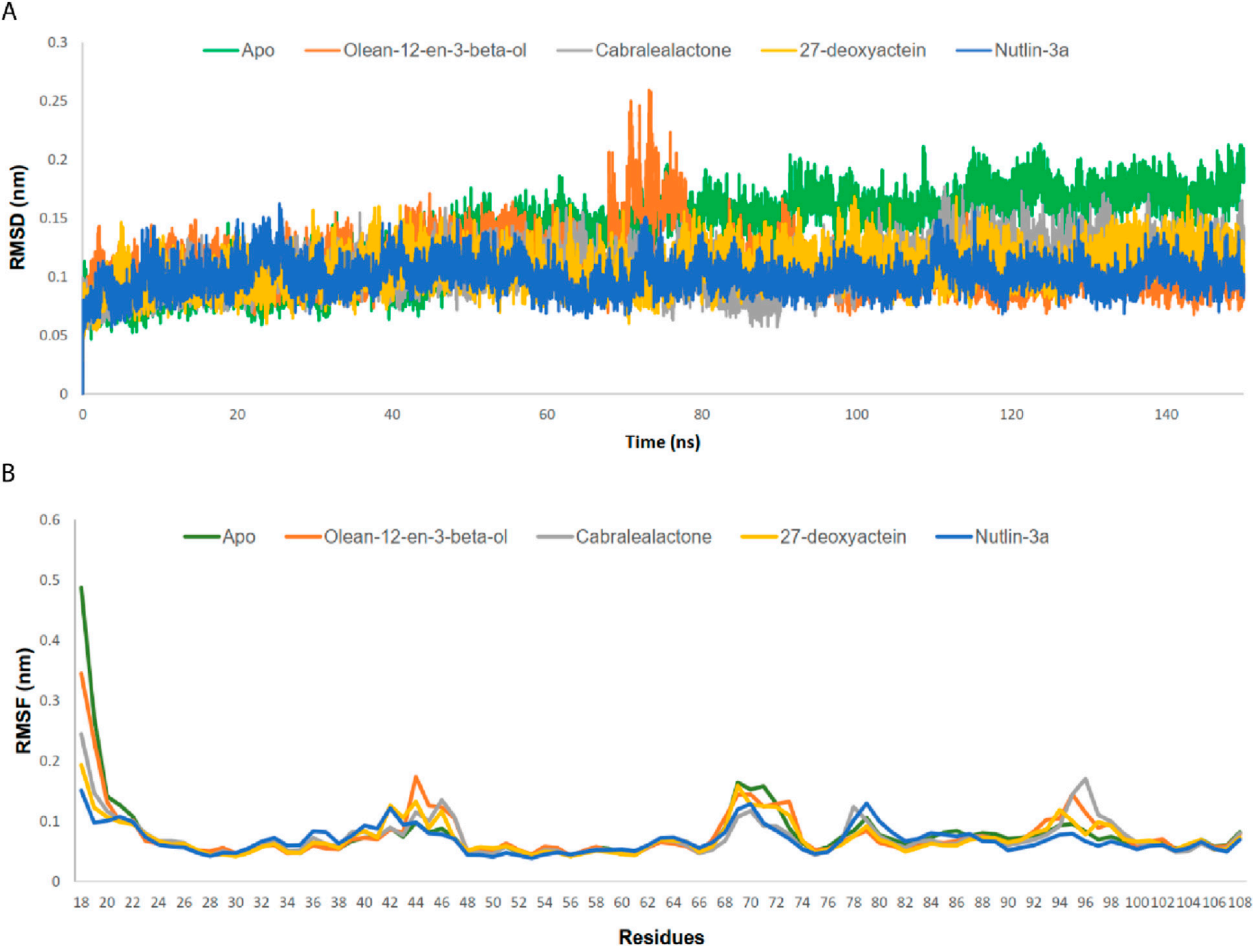
**(A)** RMSD analysis on Cα atoms of MDM2-ligand complexes. **(B)** RMSF analysis for residues of MDM2-ligand complexes.

**FIGURE 7:**
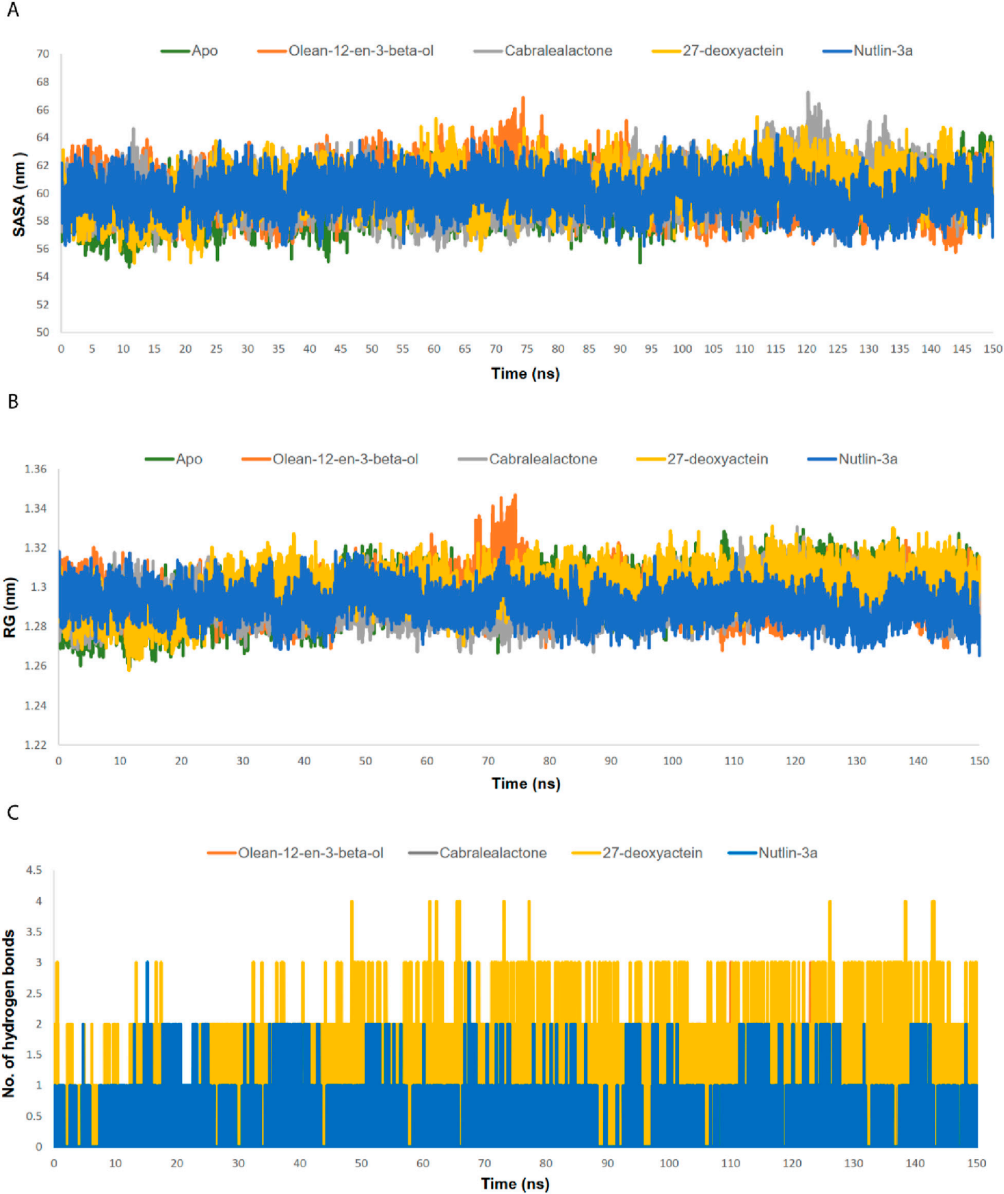
**(A)** SASA analysis of MDM2-ligand complexes. **(B)** RG analysis of MDM2-ligand complexes. **(C)** Number of hydrogen bond between MDM2 and ligands.

MD simulation plays a crucial role in post-docking analysis, allowing for the exploration of the stability and dynamic nature of biological macromolecules over time. A 150 ns MD simulation was conducted to gain insights into the structural dynamics, binding mechanisms, and flexibility of the apo state of MDM2, as well as its complexes with olean-12-en-3-beta-ol, cabralealactone, 27-deoxyactein, and Nutlin-3a. After a 150 ns dynamic trajectory, various parameters were computed and examined, including RMSD, RMSF, RG, SASA, and the number of hydrogen bonds (depicted in Figures 6, 7). These measurements were conducted as they are crucial for achieving favorable protein-ligand stabilities. Additionally, PCA and FEL analyses were carried out to investigate alterations in the conformational dynamics of the protein prior to and following ligand binding.

#### 3.5.1 RMSD and RMSF

RMSD values for the Cα backbones of all residues were calculated to confirm the stability of the apo protein and all complexes (Figure 6A). The apo protein remained stable throughout the simulation, with an average RMSD value of 0.137 nm. A slight increase in the RMSD value was observed after 48 ns, which persisted until 150 ns. All complexes exhibited lower RMSD values than the apo protein, indicating the stability of the protein-ligand complexes post-ligand binding. The average RMSD values for the olean-12-en-3-beta-ol complex, cabralealactone complex, 27-deoxyactein complex, and Nutlin-3a complex were 0.111 nm, 0.108 nm, 0.107 nm, and 0.100 nm, respectively. Although all complexes remained relatively stable throughout the simulation with minor fluctuations, a significant fluctuation in the RMSD value was observed between 70–80 ns in the case of the olean-12-en-3-beta-ol complex.

The RMSF values of both the apo protein and the protein-ligand complexes were calculated to evaluate the flexibility of the docked complexes across amino acid residues (Figure 6B). The average RMSF value for the Cα backbones of protein residues in the apo protein, olean-12-en-3-beta-ol complex, cabralealactone complex, 27-deoxyactein complex, and Nutlin-3a complex was 0.078 nm, 0.078 nm, 0.073 nm, 0.072 nm, and 0.069 nm, respectively. The N-terminal exhibited higher fluctuations, but all ligand bindings reduced the flexibility of the N-terminal as well as the overall protein flexibility. Several regions, including residues 42–47, 69–73, 78–80, and 95–97, displayed higher flexibility compared to the rest.

#### 3.5.2 SASA and RG

The SASA values of the apo protein, docked complexes, and the control were examined to comprehend alterations in the protein volume (Figure 7A). A greater SASA value suggests an expansion of the protein surface area, while a lower SASA value suggests a reduction in the protein volume. The average SASA value of the apo protein, olean-12-en-3-beta-ol complex, cabralealactone complex, 27-deoxyactein complex, and Nutlin-3a complex was 59.48 nm^2^, 60.22 nm^2^, 60.16 nm^2^, 60.28 nm^2^, and 59.89 nm^2^, respectively. As ligand binding to the active site of the protein caused a slight, negligible increase in the overall protein volume, the volume of all protein-ligand complexes remained the same as the volume of the apo state of the protein.

The RG profile was examined to evaluate the flexibility of the apo protein as well as protein-ligand complexes (Figure 7B). An elevated RG profile indicates greater flexibility attributed to the protein’s folding or unfolding mechanism. The average RG value of the apo protein, olean-12-en-3-beta-ol complex, cabralealactone complex, 27-deoxyactein complex, and Nutlin-3a complex was 1.3 nm, 1.29 nm, 1.29 nm, 1.30 nm, and 1.29 nm, respectively. Ligand binding did not affect the compactness of the protein. However, an increased RG value was observed in the case of olean-12-en-3-beta-ol complex during 70–80 ns which may be related to the increased RMSD value of the complex at that same time. So, protein complexes were as compact as apo protein which aligns with the results of the SASA value.

#### 3.5.3 Number of hydrogen bonds

We additionally assessed the hydrogen bonding between the ligands and the proteins (Figure 7C). Cabralealactone exhibited the formation of zero to two hydrogen bonds with the protein. Both Olean-12-en-3-beta-ol and Nutlin-3a established zero to three hydrogen bonds with the protein. However, in the case of 27-deoxyactein, the hydrogen bond plot revealed a maximum of four hydrogen bonds, with three hydrogen bonds consistently observed. Conversely, only one stable hydrogen bond was noted in the case of Nutlin-3a.

#### 3.5.4 PCA and FEL

**FIGURE 8:**
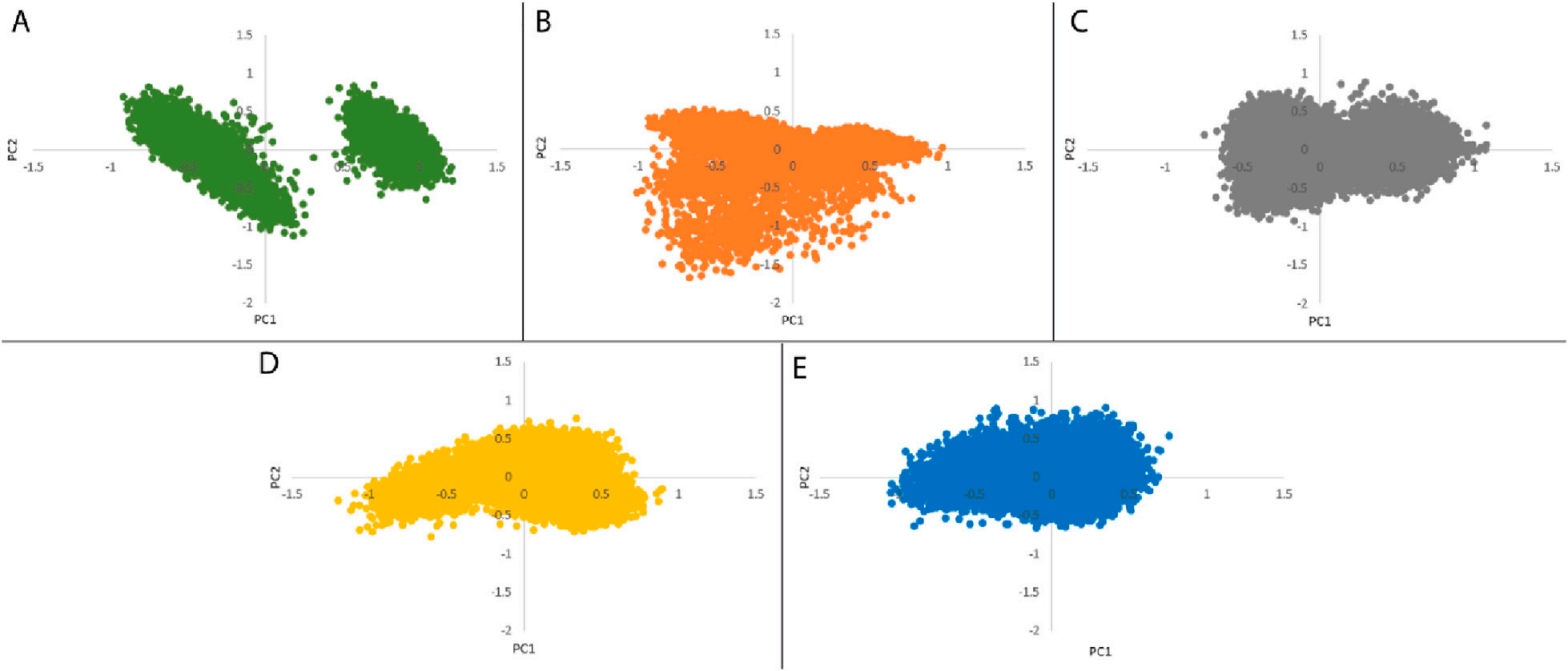
Principal component analysis of **(A)** Apo protein, **(B)** MDM2 protein-olean-12-en-3-beta-ol complex, **(C)** MDM2 protein-cabralealactone complex, **(D)** MDM2 protein-27-deoxyactein complex, and **(E)** MDM2 protein-Nutlin-3a complex.

PCA was employed to assess the stability, conformational space, and transition dynamics of both the apo protein and protein-ligand complexes by examining the relative movement of Cα atoms and the overall protein movement. The analysis resulted in clusters formed based on the projection of two eigenvectors, 1 and 2. A highly stable cluster, denoting higher stability, occupies a smaller phase space, while an unstable cluster occupies a larger space. From Figures 8A–E, 27-deoxyactein and control ligand, Nutlin-3a acquired lesser conformational space compared to the other three systems indicating their reduced motion and comparatively higher stability. Moreover, the compactness and stability of both the apo protein and protein-ligand complexes were assessed through the covariance matrix score. This evaluation revealed scores for olean-12-en-3-beta-ol complex (0.717), cabralealactone complex (0.583), 27-deoxyactein complex (0.584), and Nutlin-3a complex (0.481), indicating reduced flexibility or more restrained movements of the backbone Cα atoms in comparison to the apo state of the protein (0.784). Consequently, it can be inferred that the binding of these ligands to the protein induced stability in the protein conformations.

**FIGURE 9:**
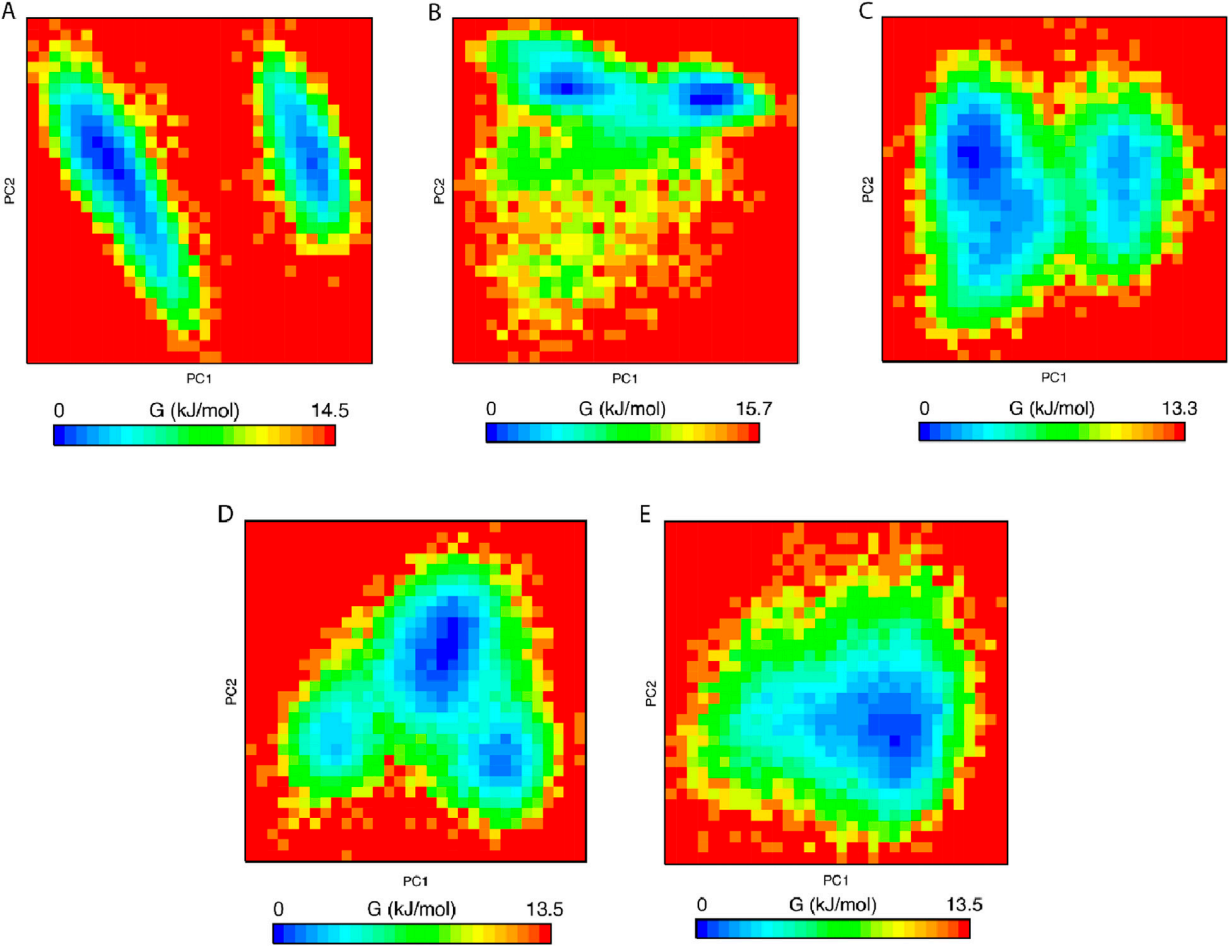
Free energy landscape of **(A)** Apo protein, **(B)** MDM2 protein-olean-12-en-3-beta-ol complex, **(C)** MDM2 protein-cabralealactone complex, **(D)** MDM2 protein-27-deoxyactein complex, and **(E)** MDM2 protein-Nutlin-3a complex.

We performed an analysis of the Gibbs free energy landscape using the first two principal components. In Figures 9A–E, the deepest blue hue indicates the protein’s conformation with the lowest energy, while the red hue indicates the conformation with the highest energy state. The profound well signifies a thermodynamically favorable state for the proteins. In this study, we have computed the free energy landscape for the apo state of MDM2 and its related protein-ligand complexes. Apo protein showed two energy minima with distinct large energy barriers which confirmed its two conformational states. On the contrary, Nutlin-3a complex showed one larger global minimum. So, it is confined to one conformational state. The rest of the three systems showed 2–3 energy minima indicating that they could acquire multiple conformational states which are separated by relativity small energy barriers. The overall energy range of the system decreased following the binding of the ligand to the protein, suggesting that these ligands formed stable and energetically favorable protein-ligand complexes with the protein.

### 3.6 MM-PBSA binding free energy calculations

**FIGURE 10:**
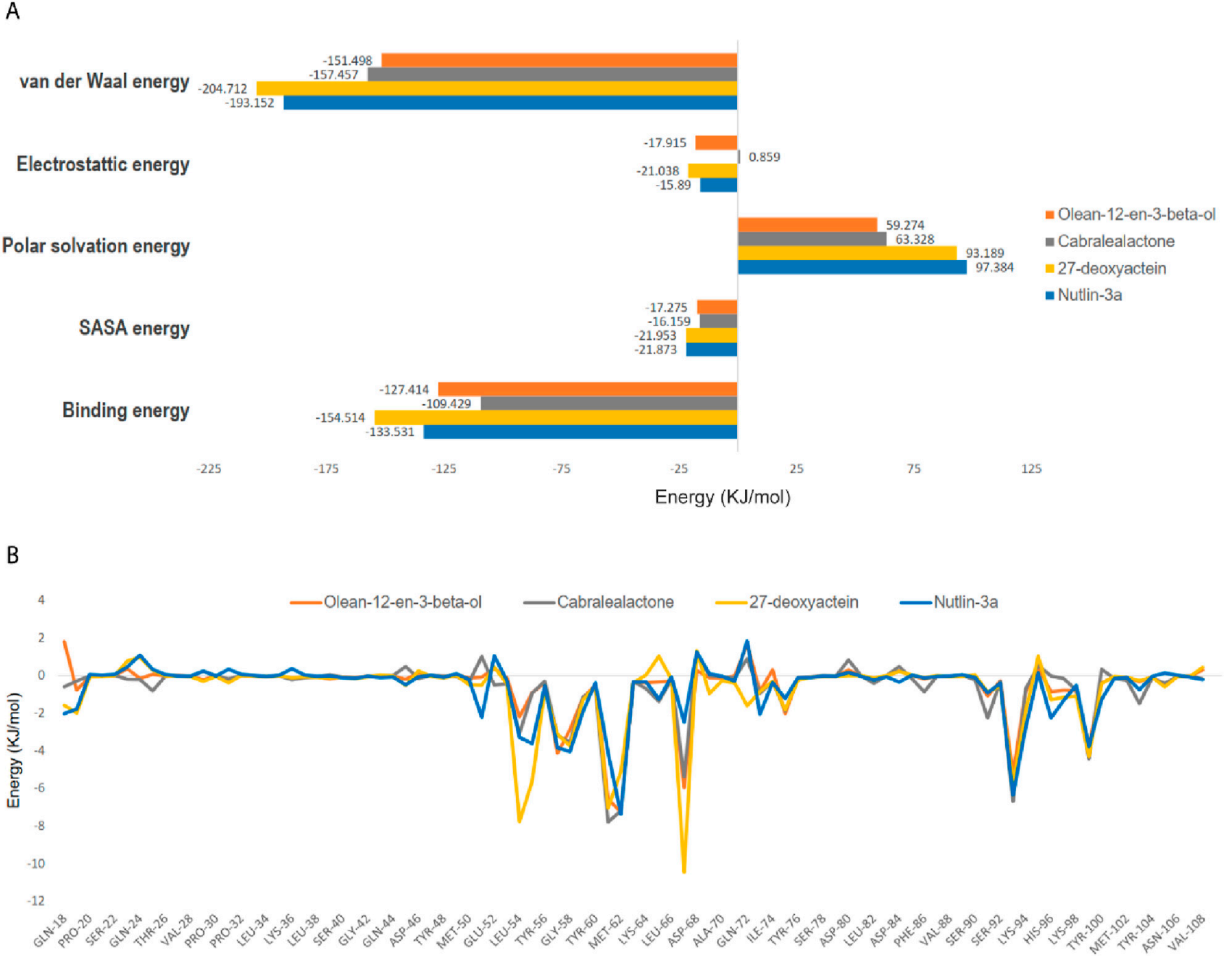
**(A)** Computed binding free energy (kJ/mol) of olean-12-en-3-beta-ol, cabralealactone, 27-deoxyactein, and Nutlin-3a with MDM2, **(B)** residues with free energy contribution of MDM2 with olean-12-en-3-beta-ol, cabralealactone, 27-deoxyactein, and Nutlin-3a. The colors represented MDM2-olean-12-en-3-beta-ol complex, MDM2-cabralealactone complex, MDM2-27-deoxyactein complex, and MDM2-Nutlin-3a complex in A and B are yellow, and green respectively.

The binding energy is governed by various molecular forces, including hydrogen bonding, van der Waals interactions, hydrophobic interactions, and electrostatic interactions between the ligand and the macromolecule. These favorable forces, in addition to solvent-accessible surface area and unfavorable polar solvation energy, were determined using MM-PBSA. The binding free energies, outlined in Table 4 and Figure 10A, were calculated using MM-PBSA methods, with more negative values indicating stronger bindings. The average binding free energies for olean-12-en-3-beta-ol, cabralealactone, 27-deoxyactein, and Nutlin-3a were – 127.414 kJ/mol, – 109.429 kJ/mol, – 154.514 kJ/mol, and – 133.531 kJ/mol, respectively. In all cases, van der Waals energy contributed the most to the binding free energy of the protein-ligand complexes. Notably, 27-deoxyactein exhibited lower binding free energy compared to other molecules and the control Nutlin-3a, suggesting its relatively better and favorable binding with the protein. Additionally, the olean-12-en-3-beta-ol complex displayed similar free energy compared to the control molecules, while cabralealactone showed the least binding affinity based on MM-PBSA calculations. Figure 10B showed that certain amino acids such as Met-62, Ile-61, Tyr-67, Val-93, Leu-54, Ile-99, Leu-57, Gly-58, Phe-55, Val-75, Lys-94, Gln-59, Phe-91, Ile-19, His-73 and His-96 played a major role for binding of ligand molecules to the protein in all instances.

**TABLE 4:** Van der Waal’s, electrostatic, polar salvation, SASA as well as binding free energy of top three selected compounds and standard inhibitor, Nutlin-3a in kJ/mol presented as mean ± standard deviation.

Compound	van der waals energy	Electrostatic energy	Polar solvation energy	SASA energy	Binding free energy
Olean-12-en-3-beta-ol	−151.498 ± 12.123	−17.915 ± 8.995	59.274 ± 10.960	−17.275 ± 1.199	−127.414 ± 13.537
Cabralealactone	−157.457 ± 10.117	−0.859 ± 6.854	63.328 ± 8.936	−16.159 ± 0.950	−109.429 ± 9.316
27-deoxyactein	−204.712 ± 11.456	−21.038 ± 15.455	93.189 ± 17.477	−21.953 ± 0.922	−154.514 ± 12.211
Nutlin-3a	−193.152 ± 15.075	−15.890 ± 8.252	97.384 ± 12.384	−21.873 ± 1.239	−133.531 ± 14.477

## 4 Discussion

BC’s varied pathology, clinical behavior, histologic classifications, and reactions to anticancer treatments complicate its safe and effective management (Lamichhane et al., 2023). The CADD approach offers a swift and cost-efficient method to investigate the anticancer properties of diverse natural compounds for the development of lead candidates aimed at combating cancer (Ahmed et al., 2023). This investigation utilized a comprehensive computational strategy to assess the anti-breast cancer potential targeting the MDM2 protein among 398 terpenoids sourced from the NPACT database. Following Lipinski’s Ro5 filtration, a two-stage screening process was employed. Initially, the terpenoid compounds were docked into the rigid crystal structure of the MDM2 protein’s active site. Then, the top-docked compounds from the initial screening were subjected to docking into the ensemble conformations of the MDM2 structure, generated through MD simulation, to capture the dynamic nature of the protein’s binding site. Three terpenoid compounds, olean-12-en-3-beta-ol, 27-deoxyactein, and cabralealactone, displayed considerable binding affinities towards the MDM2 protein and showcased favorable PK and safety profiles. However, findings from MD simulation and MM-PBSA binding free energy calculations unveiled that compound 27-deoxyactein, one of the primary components extracted from *Cimicifuga racemosa* (Suh et al., 2018), exhibited enhanced stability of the protein-ligand complex and demonstrated the lowest binding free energy in comparison to both the standard inhibitor, Nutlin-3a, and the other lead compounds.

MDM2 is an oncoprotein that interacts with the tumor suppressor protein p53, sometimes referred to as the “guardian of the genome,” to regulate the cell cycle and programmed cell death (Iwakuma and Lozano, 2003). MDM2 was reported to contribute to BC progression, invasion, and therapy resistance in both p53-dependent and -independent ways (Karni-Schmidt et al., 2016). A group of MDM2 inhibitors, including RG7112, idasanutlin, AMG-232 (KRT-232), APG-115, siremadlin (HDM201), milademetan, and CGM097, are presently undergoing clinical studies for solid tumors (Konopleva et al., 2020). Though these molecules showed significant efficacy in phase 1 or 2 trials, there are still obstacles to overcome such as possible toxicity related to the bone marrow, such as neutropenia, febrile neutropenia, and thrombocytopenia as well as gastrointestinal toxicity like nausea, vomiting, and diarrhea that may be dose-limiting (Khurana and Shafer, 2019). Additionally, there is a chance of hematopoietic defects triggering a cascade of signaling events (Kojima et al., 2005) and also developing resistance (Cinatl et al., 2014). Hence, the study we conducted strives to identify novel MDM2 inhibitors for BC to improve therapy efficacy, combat resistance, and reduce side effects, ultimately offering patients more individualized and efficient therapeutic options.

MDM2 comprises of multiple domains such as a C-terminal RING (Really Interesting New Gene) domain, an N-terminal domain, as well as a central acidic domain and each domain has distinct functionality. Its interaction with the P53 depends on the availability of a p53-binding site in the N-terminal domain. The N-terminal region of MDM2 harbors a well-defined p53-binding pocket. The RING domain acts as the active site for MDM2’s E3 ubiquitin ligase activity. In this region, MDM2 catalyzes the transfer of ubiquitin molecules to specific lysine residues on p53. This ubiquitination marks p53 for proteasomal degradation, regulating its cellular levels (Moll and Petrenko, 2003). Previous research has identified three distinct sub-pockets within the hydrophobic cleft of the MDM2 protein, each playing a crucial role in the binding to the transactivation domain of p53. These sub-pockets create an environment that accommodates specific amino acid residues of p53, disrupting the dimerization of MDM2 and p53 (Murray and Gellman, 2007). The first sub-pocket, comprising amino acids Ile-61, Met-62, and Val-93, forms the vicinity of the Phe-19 residue in p53. The second region, composed of Leu-57, Gly-58, and Ile-99, accommodates the Trp-23 residue in p53. Lastly, the Leu-54 and His-96 residues correspond to the Leu26 sub-pocket. It's noteworthy that the binding of small molecule ligands to any of these sub-pockets has the potential to interfere with the MDM2-p53 dimerization, presenting a potential therapeutic avenue for modulating the MDM2-p53 interaction (Munisamy et al., 2021; Chukwuemeka et al., 2022; Oyedele et al., 2023).

In this study, we initially conducted traditional molecular docking, where the protein was maintained as a rigid structure while the ligand was allowed to be flexible. From our library of terpenoid compounds, we identified eight novel compounds that exhibited significantly higher binding affinities than the standard inhibitor, Nutlin-3a. The range of binding energies for these top-docked terpenoid compounds was between – 8.6 kcal/mol and – 8.3 kcal/mol. Among them, cabralealactone (– 8.6 kcal/mol) and 27-deoxyactein (– 8.6 kcal/mol) exhibited the highest binding affinity toward the MDM2 protein, followed by olean-12-en-3-beta-ol (– 8.5 kcal/mol). Each of these top-docked compounds exhibited multiple non-bonding interactions with the binding site residues of the MDM2 protein, indicating their potential as novel MDM2 inhibitors. Following our preliminary molecular docking investigations, which yielded insightful information about the binding interactions between compounds of interest and the target protein, we came to understand that incorporating protein flexibility into the analyses would enhance the precision of our results. Hence, we expanded our research to include ensemble docking, a method that takes into account several protein conformations to represent the dynamic character as well as structural flexibility of the binding site. Ensemble docking investigations were carried out for the eight compounds that exhibited the highest docking scores from the initial docking studies. These compounds were docked against the top three conformers obtained from a 250 ns MD simulation of the MDM2 protein. Among these eight compounds, olean-12-en-3-beta-ol, 27-deoxyactein, and cabralealactone emerged as the top three compounds exhibiting superior binding affinities and favorable non-covalent molecular interactions (Supplementary Table S4). These interactions are indispensable for building and safeguarding the integrity of docking complexes. Resilient conventional hydrogen bonds significantly improve the overall stability of ligand-receptor complexes and are necessary for molecular recognition (Shikder et al., 2021). 27-deoxyactein was identified to form significant interactions within the binding region of the p53 and MDM2 proteins in the initial docking investigation. It specifically made conventional hydrogen bonds with Val-93 and His-96, forming crucial connections. Furthermore, hydrophobic interactions involving Val-75, Val-93, Ile-61, and Phe-91 were observed, which improved the ligand’s stability inside the crucial binding region even more. Furthermore, in ensemble docking, 27-deoxyactein was found to form one conventional hydrogen bond with Gly-58 in cluster-1 and one with Thr-49 in cluster-2. Notably, this compound demonstrated its significance by consistently forming and maintaining hydrophobic bonds with active site amino acids, including Leu-54, Val-93, Ile-99, and Ile-61 across all clusters. These findings reinforced the ligand’s potential as a promising candidate for further exploration in drug development, as it exhibited consistent and favorable interactions across different protein conformations in the ensemble. On the other hand, compounds such as olean-12-en-3-beta-ol and cabralealactone displayed comparatively similar binding patterns in the initial docking analysis. Specifically, both compounds formed conventional hydrogen bonds with Gln-24. Cabralealactone further established hydrophobic bonds with critical amino acid residues, including Leu-54, Val-93, Ile-61, Met-62, and Lys-51. Similarly, olean-12-en-3-beta-ol engaged in hydrophobic interactions with Val-93, Leu-54, Ile-19, Ile-61, His-96, and Tyr-100. During ensemble docking, olean-12-en-3-beta-ol showed an intriguing binding pattern by not forming hydrogen bonds with the first two clusters. However, it notably established conventional hydrogen bonds with the active site amino acid residue Tyr-100 in cluster-3. This observation suggested a specific molecular interaction contributing to the higher binding affinities observed within cluster-3. Nevertheless, olean-12-en-3-beta-ol consistently formed hydrophobic bonds with key active site residues, including Leu-54, Ile-99, Met-62, and Val-93, across all three clusters. This consistent interaction profile underscored the ligand’s capability to contribute to the stability of the ligand-receptor complex, further emphasizing its potential as a valuable candidate for drug development. However, in the case of cabralealactone, despite forming a hydrogen bond with Gln-72 in cluster-1, this interaction was not observed in the other two clusters. However, the molecule consistently formed hydrophobic interactions with critical residues of amino acids, including Leu-54, Ile-61, and Val-93, in each of the three clusters. The compound’s stability and potential as a promising ligand were highlighted by its persistent hydrophobic interaction profile, which also illuminated the underlying causes for its greater binding affinities.

Servers for computational ADME and toxicity evaluations have dramatically enhanced recenlty, which allowed for swift analyses to assess numerous pharmacokinetic, pharmacodynamic as well as toxicity characteristics of drug candidates (Tao et al., 2015). In this study, a comprehensive analysis of selected top three chemical compounds, olean-12-en-3-beta-ol, 27-deoxyacetein, and cabralealactone were studied to understand their attributes and suitability as drug candidates. Absorption is a critical parameter influencing a drug’s bioavailability and therapeutic efficacy. Compounds with high GI absorption, such as 27-deoxyacetein and cabralealactone, are more likely to reach systemic circulation in sufficient quantities to exert therapeutic effects. In contrast, Olean-12-en-3-beta-ol exhibits lower GI absorption, which may necessitate higher doses or alternative delivery strategies to achieve therapeutic concentrations. Moreover, all three lead compounds have good bioavailability scores. The BBB protects the brain from harmful substances by permitting only certain substances to pass from the bloodstream into the brain (Wu et al., 2023). None of the three compounds cross the BBB, so the risk of CNS adverse effects is greatly reduced. The metabolism of medications and foreign substances depends on cytochrome p50 enzymes. Specifically, the most important cytochrome p50 enzymes are CYP3A4 and CYP2D6 (Lynch and Price, 2007). Our three selected phytochemicals do not inhibit CYP3A4 or CYP2D6. PAINS criterion revealed zero alerts for all three selected terpenoid compounds indicating that these leads would not cause false positive results (Baell and Nissink, 2018). Moreover, 27-Deoxyacetein demonstrated a high synthetic accessibility value, while the remaining two lead compounds displayed a moderate level of synthetic accessibility. As a result, all three compounds can be easily synthesized. Moreover, the toxicity assessments yielded favorable outcomes, showing no significant adverse effects across multiple toxicity endpoints and parameters. This further supports our selection of the top three lead candidates. However, it's essential to recognize that predictive toxicity assessments have limitations and should be complemented with experimental data to validate safety profiles accurately.

Several MDM2 inhibitors have been identified from terpenoid compounds, including Celastrol, Triptolide, Withaferin A, Costunolide, and Betulinic Acid (Cascão et al., 2017; Zhao et al., 2022; Ramli et al., 2024). However, despite their anticancer potential, many of these compounds possess reactive functional groups and exhibit poor pharmacokinetics, limiting their clinical utility. For instance, Celastrol contains a highly reactive quinone methide moiety, which contributes to off-target toxicity and metabolic instability. Its poor aqueous solubility further hampers bioavailability (Shi et al., 2020). Similarly, Triptolide harbors an epoxide group that predisposes it to non-specific protein binding, leading to undesirable side effects and restricted clinical applicability (Ren et al., 2021).

In contrast, Olean-12-en-3-beta-ol, 27-Deoxyactein, and Cabralealactone offer distinct structural advantages that enhance their pharmacological viability. These compounds are more structurally stable due to the absence of highly reactive moieties, ensuring greater metabolic stability and reduced off-target toxicity. Their functional groups, such as hydroxyl (-OH) and lactone (-COO-), promote hydrogen bonding while maintaining hydrophobic interactions essential for strong MDM2 binding (Ishmuratov et al., 2015). Olean-12-en-3-beta-ol possesses a pentacyclic triterpenoid backbone, optimizing its fit within the MDM2 binding pocket while minimizing steric hindrance, a challenge faced by bulkier polar compounds such as Ganoderic Acid A (Jia et al., 2023). 27-Deoxyactein, with its steroidal framework, enhances binding efficiency by maintaining optimal molecular rigidity, overcoming the flexibility issues seen in Japonicone A and Parthenolide, which have sesquiterpene lactone structures that lower binding stability (Schmidt, 2006). Cabralealactone stands out due to its rigid bicyclic lactone core, ensuring superior geometric alignment within the binding site compared to structurally flexible counterparts. Unlike Costunolide and Parthenolide, whose lactone moieties exhibit high conformational flexibility, Cabralealactone’s rigid structure enhances its binding affinity and selectivity (Zhang et al., 2023). Furthermore, these newly identified terpenoids share beneficial pharmacophoric features with previously studied compounds while offering distinct advantages. The hydrophobic interactions of Olean-12-en-3-beta-ol, 27-Deoxyactein, and Cabralealactone resemble those of Ganoderic Acid A, ensuring stable MDM2 binding. However, their structural rigidity prevents steric clashes, improving binding efficiency. The hydroxyl (-OH) and lactone (-COO-) groups, also found in Withaferin A and Betulinic Acid, contribute to selective and stable interactions while enhancing metabolic stability. Notably, the lactone moiety in Cabralealactone mirrors those in Costunolide and Parthenolide, yet its more rigid framework provides superior binding alignment, reducing conformational entropy loss upon binding.

These structural and pharmacokinetic advantages position Olean-12-en-3-beta-ol, 27-Deoxyactein, and Cabralealactone as more selective, metabolically stable, and pharmacologically viable MDM2 inhibitors compared to previously studied natural terpenoids. Their improved binding efficiency, reduced reactivity, and enhanced pharmacokinetic profiles advance current research on MDM2 inhibition, offering promising candidates for breast cancer therapy.

MD simulations are essential for grasping the dynamic behavior of macromolecules, including their structural stability and degree of flexibility. Various parameters such as RMSD, RMSF, RG, SASA, the number of hydrogen bonds, PCA, and FEL are employed to assess the dynamic characteristics of biological macromolecules and protein-ligand complexes (Roe and Cheatham III, 2013). Together, these parameters reveal the intricate relationship between stability and structural dynamics, providing vital knowledge for comprehending bio-molecular interactions and directing the logical development of drugs.

RMSD was analyzed to observe the overall stability of apo protein and protein-ligand complexes. From the result, it is observed that all the complexes achieved stability in their 150 ns MD simulation except for a little deviation of the olean-12-en-3-beta-ol complex. However, 27-deoxyactein complex was more stable along with Nutlin-3a complex compared to apo and the rest of the two complexes. RMSF was calculated to analyze the flexibility of the backbone atoms of the residues in apo-protein and protein-ligand complexes. Except for the N-terminal of the protein, all the complexes show almost the same RMSF values. Upon ligand binding, the flexibility of the protein reduces initially compared to apo-protein but in the other region, these complexes show almost similar flexibility. However, 27-deoxyactein complex showed comparatively less fluctuation among the apo protein and other complexes along with Nutlin-3a complex, thus confirming 27-deoxyactein’ well-fitting in the active site of the protein and forming a stable complex. So, RMSD and RMSF values comply with each other in favor of the stability of 27-deoxyactein complex.

SASA value mainly clarifies a protein’s solvent behavior, specifically if it demonstrates hydrophilic or hydrophobic characteristics. It took into account interactions between atoms that are both polar and non-polar. Since SASA represents the middle part of the solvent molecules surrounding the receptor molecule, it was used as a metric to assess changes in the protein’s structure. In our investigations, SASA was slightly increased after the ligand binding to the protein. This phenomenon was observed in the complexes of three lead compounds as well as in the complex formed by the standard inhibitor, Nutlin-3a. This could happen because these ligands occupy the active site of the protein, primarily located within the large hydrophobic cleft of the MDM2 protein. Moreover, RG was analyzed to investigate the compactness of the protein upon binding to ligands. The RG value of was Nutlin-3a complex was 1.2909 nm whereas the apo-protein showed an RG value of 1.2961 nm. All protein-ligand complexes, including the 27-deoxyactein complex (1.2989 nm), the olean-12-en-3-beta-ol complex (1.2949 nm), and the cabralealactone complex (1.2919 nm), exhibited a close alignment with the apo-protein. Hence, it is observed that the compactness of all protein-ligand complexes remained almost the same as apo-protein.

Hydrogen bonds formed between the ligands and the protein throughout the simulation are assessed using the *gmx hbond* utility. The highest average number of hydrogen bonds was observed in the 27-deoxyactein complex (1.1349), which was significantly greater than that in the standard inhibitor, Nutlin-3a complex (0.3374). Other complexes, such as olean-12-en-3-beta-ol and cabralealactone, formed an average of 0.4543 and 0.0264 hydrogen bonds, respectively. The number of intermolecular hydrogen bonds was particularly prominent in the 27-deoxyactein complex, and these hydrogen bonds remained stable throughout the simulation, indicating the formation of a more stable complex between 27-deoxyactein and the MDM2 protein compared to the other complexes.

PCA assessed the stability, conformational space, and transition dynamics of apo protein and protein-ligand complexes via Cα atoms’ relative movement and overall protein motion. From the result, 27-deoxyactein complex and the Nutlin-3a complex acquired less conformational space and had a lower covariance matrix score than the other three systems including apo protein which proved the superiority of these two compounds based on their stability and compactness over others. This is due to the binding of ligands to the active site of the protein which restricted the movement and reduced flexibility of the backbone of Cα atoms of the protein. FELs offer a thorough understanding of the thermodynamic features of biomolecular systems, including energies, folding processes, and stability. The mechanisms driving biological activities including protein folding, ligand binding, and molecular recognition can be understood in light of this information. From FELs, it is observed that the apo protein showed two conformational states, whereas the Nutlin-3a possessed only one conformation. But the rest of the system including 27-deoxyactein showed several stable conformations. This is due to the strong bond formation of these compounds to MDM2 and changing the folding of the protein chain to several energetically favorable distinguish structures.

MM-PBSA binding free energy calculation is a widely used technique to validate molecular docking results. When comparing the binding energies found using this method to the scores produced by AutoDock Vina, the former turned out to be more trustworthy. The spontaneous nature of the binding of all complexes tested was confirmed by the observation of negative total binding energies. Intermolecular Vander Waals energy, electrostatic energy, SASA energy, and polar solvation energy make up a complex’s binding free energy. 27-deoxyactein had lower binding energy (– 154.514 kJ/mol) compared to Nutlin-3a (– 133.531 kJ/mol). Indeed, Vander Waals energy, electrostatic energy, and SASA energy favored the binding of 27-deoxyactein to MDM2 than the standard inhibitor, Nutlin-3a to MDM2. Moreover, the increased number of hydrogen bonds and their sustainability further confirm the findings. Major contributing amino acids of 27-deoxyactein complex in the bond energy was Leu-54 (– 7.76 kJ/mol), Ile-61 (– 7.05 kJ/mol), Val-93 (– 5.71 kJ/mol), Met-62 (– 5.10 kJ/mol), Ile-99 (– 4.30 kJ/mol), Gly-58 (– 3.75 kJ/mol) and Leu-57 (– 3.12 kJ/mol). These amino acids’ positions are in the active site of the protein, thus confirming the binding of 27-deoxyactein to the active place of the MDM2.

Analyzing these parameters, it can be concluded that the MDM2-27-deoxyactein complex was the most stable, where 27-deoxyactein bind to the active site of the targeted protein MDM2 with sufficient binding affinities. Among all the compounds, it was the most potent compound for effectively inhibiting MDM2. It also showed favorable ADME and toxicity attributes to be a potential drug candidate. Hence, the natural terpenoid compound, 27-deoxyactein, holds promise as a strong candidate for inhibiting MDM2, warranting further investigation as a lead compound in experimental and clinical studies to enhance breast cancer treatment efforts.

Our findings contribute to current breast cancer research by identifying novel, pharmacologically viable natural terpenoid inhibitors that target MDM2 with greater stability and selectivity than previously studied compounds. While MDM2 inhibitors have been explored for BC therapy, many face challenges such as metabolic instability, off-target effects, and poor pharmacokinetics, limiting their clinical application (Ramli et al., 2024). By addressing the limitations of existing inhibitors, our study provides a strong computational foundation for experimental validation, advancing the search for effective MDM2-targeted therapies to overcome BC progression and drug resistance. Computational methods play a crucial role in real-world drug development by accelerating the discovery of potential drug candidates, reducing costs, and improving success rates. These methods help prioritize compounds with high therapeutic potential, streamlining lead optimization and reducing reliance on costly and time-consuming laboratory screening. However, Experimental validation is essential to confirm the therapeutic potential of these inhibitors, yet it presents its own challenges. *In vitro* studies, though useful for preliminary screening, fail to replicate the complexity of the tumor microenvironment, and *in vivo* models introduce variability and ethical concerns. Furthermore, terpenoid metabolism and long-term safety must be thoroughly assessed, as rapid metabolism can diminish efficacy. Therefore, more effective bioassays need to be developed to assess and facilitate the translation of computational results into clinical settings.

## 5 Conclusion

To sum up, breast cancer (BC) remains the leading cause of cancer-related deaths in women, with MDM2, a critical inhibitor of the tumor suppressor p53, playing a central role in its progression. This study utilized computational approaches to explore the potential of natural terpenoid compounds as novel inhibitors of MDM2. Initially, rigid protein-flexible ligand molecular docking identified eight compounds with higher binding affinities than the standard inhibitor Nutlin-3a. These compounds were further evaluated using ensemble docking with multiple MDM2 conformations derived from a 250 ns MD simulation. Among the compounds tested, olean-12-en-3-beta-ol, 27-deoxyactein, and cabralealactone showed significantly high binding affinities across various conformations. ADMET analysis confirmed favorable pharmacokinetic and pharmacodynamic properties for all three, with minimal side effects. Additionally, these lead compounds demonstrated substantial stability during 150 ns MD simulations. Notably, 27-deoxyactein emerged as the most promising candidate according to MM-PBSA analysis, showing superior binding stability. However, while computational findings are promising, we recommend further *in vitro* and *in vivo* investigations to experimentally validate these results and assess their real-world therapeutic potential.

## Data Availability

The original contributions presented in the study are included in the article/Supplementary Material, further inquiries can be directed to the corresponding authors.
