# Supplementary material for: Computational identification of potential natural terpenoid inhibitors of MDM2 for breast cancer therapy: molecular docking, molecular dynamics simulation, and ADMET analysis
Source: Front Chem. 2025 Apr 16;13:1527008. doi: 10.3389/fchem.2025.1527008 (PMC12041027; doi:10.3389/fchem.2025.1527008)
Supplement: Supplementary file 1 [file Table1.docx]

***Supplementary Material***

**TABLE S1** Lipinski rule of five screening and molecular docking result of 398 terpenoid compound against MDM2 protein

| **Sl no** | **Compound Name** | **PubChem ID** | **SMILES** | **Lipinski rule of five** | **Binding Score (kcal/mol)** |
| --- | --- | --- | --- | --- | --- |
| 1 | Menthol | 1254 | CC1CCC(C(C1)O)C(C)C | yes | - 5 |
| 2 | dl-a-Tocopherol | 2116 | CC1=C(C2=C(CCC(O2)(C)CCCC(C)CCCC(C)CCCC(C)C)C(=C1O)C)C | yes | - 6.4 |
| 3 | camphor | 2537 | CC1(C2CCC1(C(=O)C2)C)C | Yes | - 5.1 |
| 4 | Cineole | 2758 | CC1(C2CCC(O1)(CC2)C)C | yes | - 5 |
| 5 | Eugenol | 3314 | COC1=C(C=CC(=C1)CC=C)O | yes | - 5.2 |
| 6 | Glycyrrhizic acid ammonium salt | 3495 | CC1(C2CCC3(C(C2(CCC1OC4C(C(C(C(O4)C(=O)O)O)O)OC5C(C(C(C(O5)C(=O)O)O)O)O)C)C(=O)C=C6C3(CCC7(C6CC(CC7)(C)C(=O)O)C)C)C)C | No |  |
| 7 | (-)-Gossypol | 3503 | CC1=CC2=C(C(=C(C(=C2C(C)C)O)O)C=O)C(=C1C3=C(C4=C(C=C3C)C(=C(C(=C4C=O)O)O)C(C)C)O)O | No |  |
| 8 | bornyl acetate | 6448 | CC(=O)OC1CC2CCC1(C2(C)C)C | Yes | - 5.4 |
| 9 | Linalool | 6549 | CC(=CCCC(C)(C=C)O)C | yes | - 5.2 |
| 10 | Camphene | 6616 | CC1(C2CCC(C2)C1=C)C | yes | - 4.8 |
| 11 | Alpha-Pinene | 6654 | CC1=CCC2CC1C2(C)C | Yes | - 5.2 |
| 12 | Menthone | 6986 | CC1CCC(C(=O)C1)C(C)C | yes | - 5.2 |
| 13 | Pulegone | 6988 | CC1CCC(=C(C)C)C(=O)C1 | Yes | - 5.7 |
| 14 | Thymol | 6989 | CC1=CC(=C(C=C1)C(C)C)O | yes | - 5.3 |
| 15 | Carvyl acetate | 7335 | CC1=CCC(CC1OC(=O)C)C(=C)C | yes | - 5.5 |
| 16 | P-Cymene | 7463 | CC1=CC=C(C=C1)C(C)C | yes | - 5.5 |
| 17 | Citronellal | 7794 | CC(CCC=C(C)C)CC=O | yes | - 5.1 |
| 18 | Citronellyl propionate | 8834 | CCC(=O)OCCC(C)CCC=C(C)C | yes | - 5.6 |
| 19 | Citronellol | 8842 | CC(CCC=C(C)C)CCO | yes | - 4.9 |
| 20 | 18-beta-Glycyrrhetinic acid | 10114 | CC1(C2CCC3(C(C2(CCC1O)C)C(=O)C=C4C3(CCC5(C4CC(CC5)(C)C(=O)O)C)C)C)C | Yes | - 7.6 |
| 21 | Carvacrol | 10364 | CC1=C(C=C(C=C1)C(C)C)O | yes | - 5.6 |
| 22 | Oleanolic acid | 10494 | CC1(CCC2(CCC3(C(=CCC4C3(CCC5C4(CCC(C5(C)C)O)C)C)C2C1)C)C(=O)O)C | yes | - 7.7 |
| 23 | Myrtenol | 10582 | CC1(C2CC=C(C1C2)CO)C | yes | - 4.9 |
| 24 | Bisabolene | 10586 | CC1=CCC(CC1)C(C)(CCC=C(C)C)O | yes | - 6.8 |
| 25 | Perillyl alcohol | 10819 | CC(=C)C1CCC(=CC1)CO | yes | - 5.5 |
| 26 | beta-phellandrene | 11142 | CC(C)C1CCC(=C)C=C1 | Yes | - 5.4 |
| 27 | terpinolene | 11463 | CC1=CCC(=C(C)C)CC1 | Yes | - 5.7 |
| 28 | beta-caryophyllene 4,5 alpha-oxide | 14350 | CC1(CC2C1CCC3(C(O3)CCC2=C)C)C | Yes | - 5.5 |
| 29 | Fenchone | 14525 | CC1(C2CCC(C2)(C1=O)C)C | yes | - 5 |
| 30 | damsin | 14631 | CC1CCC2C(C3(C1CCC3=O)C)OC(=O)C2=C | Yes | - 6.2 |
| 31 | beta-pinene | 14896 | CC1(C2CCC(=C)C1C2)C | Yes | - 5.2 |
| 32 | Glycyrrhizic acid | 14982 | CC1(C2CCC3(C(C2(CCC1OC4C(C(C(C(O4)C(=O)O)O)O)OC5C(C(C(C(O5)C(=O)O)O)O)O)C)C(=O)C=C6C3(CCC7(C6CC(CC7)(C)C(=O)O)C)C)C)C | No |  |
| 33 | Fenchol | 15406 | CC1(C2CCC(C2)(C1O)C)C | yes | - 4.9 |
| 34 | Perillaldehyde | 16441 | CC(=C)C1CCC(=CC1)C=O | yes | - 5.3 |
| 35 | Alpha-Terpineol | 17100 | CC1=CCC(CC1)C(C)(C)O | yes | - 5.3 |
| 36 | Limonene | 22311 | CC1=CCC(CC1)C(=C)C | Yes | - 5.4 |
| 37 | helenalin | 23205 | CC1CC2C(C(C3(C1C=CC3=O)C)O)C(=C)C(=O)O2 | Yes | - 6.3 |
| 38 | Myrcene | 31253 | CC(=CCCC(=C)C=C)C | yes | - 5.1 |
| 39 | Paclitaxel | 36314 | CC1=C2C(C(=O)C3(C(CC4C(C3C(C(C2(C)C)(CC1OC(=O)C(C(C5=CC=CC=C5)NC(=O)C6=CC=CC=C6)O)O)OC(=O)C7=CC=CC=C7)(CO4)OC(=O)C)O)C)OC(=O)C | No |  |
| 40 | 8-epi-helenalin | 52167 | CC1CC2C(C(C3(C1C=CC3=O)C)O)C(=C)C(=O)O2 | Yes | - 6.5 |
| 41 | borneol | 64685 | CC1(C2CCC1(C(C2)O)C)C | Yes | - 4.7 |
| 42 | Ursolic acid | 64945 | CC1CCC2(CCC3(C(=CCC4C3(CCC5C4(CCC(C5(C)C)O)C)C)C2C1C)C)C(=O)O | Yes | - 8 |
| 43 | Betulinic acid | 64971 | CC(=C)C1CCC2(C1C3CCC4C5(CCC(C(C5CCC4(C3(CC2)C)C)(C)C)O)C)C(=O)O | yes | - 8.6 |
| 44 | Carnosic acid | 65126 | CC(C)C1=C(C(=C2C(=C1)CCC3C2(CCCC3(C)C)C(=O)O)O)O | yes | - 7.2 |
| 45 | Verbenone | 65724 | CC1=CC(=O)C2CC1C2(C)C | Yes | - 5.2 |
| 46 | Betulin | 72326 | CC(=C)C1CCC2(C1C3CCC4C5(CCC(C(C5CCC4(C3(CC2)C)C)(C)C)O)C)CO | yes | - 8.2 |
| 47 | 23,24-dihydrocucurbitacin F | 72421 | CC1(C(C(CC2C1=CCC3C2(C(=O)CC4(C3(CC(C4C(C)(C(=O)CCC(C)(C)O)O)O)C)C)C)O)O)C | Yes | - 7.2 |
| 48 | encelin | 72540 | CC12CC3C(CC1C(=C)C(=O)C=C2)C(=C)C(=O)O3 | Yes | - 6.5 |
| 49 | Bruceoside C | 73122 | CC1=C(C(=O)CC2(C1C(C3C45C2C(C(C(C4CC(=O)O3)(OC5)C(=O)OC)O)O)OC(=O)C=C(C)C)C)OC6C(C(C(C(O6)CO)O)O)O | No |  |
| 50 | Chaparrinone | 73154 | CC1C2CC(=O)OC3C24COC(C1O)(C4C5(C(C3)C(=CC(=O)C5O)C)C)O | yes | - 7 |
| 51 | alpha-amyrin | 73170 | CC1CCC2(CCC3(C(=CCC4C3(CCC5C4(CCC(C5(C)C)O)C)C)C2C1C)C)C | Yes | - 8.6 |
| 52 | Dehydrocostuslactone | 73174 | C=C1CCC2C(C3C1CCC3=C)OC(=O)C2=C | yes | - 6.8 |
| 53 | Alpha-hederin | 73296 | CC1C(C(C(C(O1)OC2C(C(COC2OC3CCC4(C(C3(C)CO)CCC5(C4CC=C6C5(CCC7(C6CC(CC7)(C)C)C(=O)O)C)C)C)O)O)O)O)O | No |  |
| 54 | Hederagenin | 73299 | CC1(CCC2(CCC3(C(=CCC4C3(CCC5C4(CCC(C5(C)CO)O)C)C)C2C1)C)C(=O)O)C | yes | - 7.3 |
| 55 | Maslinic acid | 73659 | CC1(CCC2(CCC3(C(=CCC4C3(CCC5C4(CC(C(C5(C)C)O)O)C)C)C2C1)C)C(=O)O)C | yes | - 7.6 |
| 56 | tricyclene | 79035 | CC1(C2CC3C1(C3C2)C)C | Yes | - 4.9 |
| 57 | Aucubin | 91458 | C1=COC(C2C1C(C=C2CO)O)OC3C(C(C(C(O3)CO)O)O)O | yes | - 5.7 |
| 58 | Gamma-Tocopherol | 92729 | CC1=C(C=C2CCC(OC2=C1C)(C)CCCC(C)CCCC(C)CCCC(C)C)O | yes | - 6.1 |
| 59 | vulgarin | 94253 | CC1C2CCC3(C(C2OC1=O)C(C=CC3=O)(C)O)C | Yes | - 6.6 |
| 60 | Nimbolide | 100017 | CC1=C2C(CC1C3=COC=C3)OC4C2(C(C5(C6C4OC(=O)C6(C=CC5=O)C)C)CC(=O)OC)C | yes | - 8.1 |
| 61 | Erythrodiol | 101761 | CC1(CCC2(CCC3(C(=CCC4C3(CCC5C4(CCC(C5(C)C)O)C)C)C2C1)C)CO)C | yes | - 8 |
| 62 | Caulophyllogenin | 104361 | CC1(CCC2(C(C1)C3=CCC4C5(CCC(C(C5CCC4(C3(CC2O)C)C)(C)CO)O)C)C(=O)O)C | yes | - 6.9 |
| 63 | Saikosaponin D | 107793 | CC1C(C(C(C(O1)OC2CCC3(C(C2(C)CO)CCC4(C3C=CC56C4(CC(C7(C5CC(CC7)(C)C)CO6)O)C)C)C)O)OC8C(C(C(C(O8)CO)O)O)O)O | No |  |
| 64 | Geniposide | 107848 | COC(=O)C1=COC(C2C1CC=C2CO)OC3C(C(C(C(O3)CO)O)O)O | yes | - 6.1 |
| 65 | Trichothecolone | 107974 | CC1=CC2C(CC1=O)(C3(C(CC(C34CO4)O2)O)C)C | Yes | - 6.6 |
| 66 | Toosendanal | 115060 | CC(=O)OC1CC(C23COC(C1(C2CC(C4(C3C(=O)C(C5(C46C(O6)CC5C7=COC=C7)C)OC(=O)C)C)O)C)O)O | No |  |
| 67 | Asiatic acid | 119034 | CC1CCC2(CCC3(C(=CCC4C3(CCC5C4(CC(C(C5(C)CO)O)O)C)C)C2C1C)C)C(=O)O | yes | - 7.6 |
| 68 | Celastrol | 122724 | CC1=C(C(=O)C=C2C1=CC=C3C2(CCC4(C3(CCC5(C4CC(CC5)(C)C(=O)O)C)C)C)C)O | yes | - 8.5 |
| 69 | Hederagenin 28-O-beta-D-glucopyranosyl ester | 127026 | CC1(CCC2(CCC3(C(=CCC4C3(CCC5C4(CCC(C5(C)CO)O)C)C)C2C1)C)C(=O)OC6C(C(C(C(O6)CO)O)O)O)C | No |  |
| 70 | Glaucocalyxin A | 127626 | CC1(C2CC(C34C(C2(CCC1=O)C)CCC(C3O)C(=C)C4=O)O)C | yes | - 7.2 |
| 71 | Glycyrrhizin | 128229 | CC1(C2CCC3(C(C2(CCC1OC4C(C(C(C(O4)C(=O)O)O)O)OC5C(C(C(C(O5)C(=O)O)O)O)O)C)C(=O)C=C6C3(CCC7(C6CC(CC7)(C)C(=O)O)C)C)C)C | No |  |
| 72 | Ludovicin C | 147182 | CC1=C2C3C(CCC2(C(CC1=O)O)C)C(=C)C(=O)O3 | Yes | - 7 |
| 73 | P-Mentha-2,8-dien-1-ol | 155626 | CC(=C)C1CCC(C=C1)(C)O | yes | - 5.1 |
| 74 | Pristimerin | 159516 | CC1=C(C(=O)C=C2C1=CC=C3C2(CCC4(C3(CCC5(C4CC(CC5)(C)C(=O)OC)C)C)C)C)O | yes | - 8.2 |
| 75 | 28-deoxonimbolide | 159573 | CC1=C2C(CC1C3=COC=C3)OC4C2(C(C5(C6C4OCC6(C=CC5=O)C)C)CC(=O)OC)C | Yes | - 7.3 |
| 76 | Hederagenin 3-O-alpha-L-arabinopyranoside | 159813 | CC1(CCC2(CCC3(C(=CCC4C3(CCC5C4(CCC(C5(C)CO)OC6C(C(C(CO6)O)O)O)C)C)C2C1)C)C(=O)O)C | Yes | - 8.1 |
| 77 | xanthinin | 160533 | CC1CC2C(CC=C1C(CC(=O)C)OC(=O)C)C(=C)C(=O)O2 | Yes | - 7 |
| 78 | T-cadinol | 160799 | CC1=CC2C(CCC(C2CC1)(C)O)C(C)C | Yes | - 6.2 |
| 79 | Paucin | 161538 | CC1CC2C(CC3(C1C(CC3=O)OC4C(C(C(C(O4)COC(=O)C)O)O)O)C)C(=C)C(=O)O2 | Yes | - 7.1 |
| 80 | Sapindoside B | 161686 | CC1C(C(C(C(O1)OC2C(C(COC2OC3CCC4(C(C3(C)CO)CCC5(C4CC=C6C5(CCC7(C6CC(CC7)(C)C)C(=O)O)C)C)C)O)O)O)OC8C(C(C(CO8)O)O)O)O | No |  |
| 81 | deacetoxymatricarin | 167683 | CC1C2CCC(=C3C(C2OC1=O)C(=CC3=O)C)C | Yes | - 6.8 |
| 82 | Ludovicin A | 168722 | CC12CCC3C(C1C4(C(O4)CC2O)C)OC(=O)C3=C | Yes | - 6.6 |
| 83 | Ludovicin B | 168723 | CC12CCC3C(C1C(=C)C(CC2O)O)OC(=O)C3=C | Yes | - 6.6 |
| 84 | Cussonoside B | 169187 | CC1C(C(C(C(O1)OC2C(OC(C(C2O)O)OCC3C(C(C(C(O3)OC(=O)C45CCC(CC4C6=CCC7C8(CCC(C(C8CCC7(C6(CC5)C)C)(C)C)O)C)(C)C)O)O)O)CO)O)O)O | No |  |
| 85 | arteglasin A | 169494 | CC1=C2CC3C(C2C4C(C(C1)OC(=O)C)C(=C)C(=O)O4)(O3)C | Yes | - 7.4 |
| 86 | Meliatoxin B1 | 174647 | CCC(C)C(=O)OC1C2(C3CC(C4(C5CC(=O)C(C5(CC(=O)C4C3(CO1)C(C(C2OC(=O)C)OC(=O)C)O)C)C6=COC=C6)C)O)C | No |  |
| 87 | Curcusone A | 175941 | CC1CC2=C(C1=O)C3C(C=C(C2=O)C)C(CCC3=C)C(=C)C | yes | - 7.7 |
| 88 | Curcusone C | 175942 | CC1=CC2C(CCC(=C)C2C3=C(C1=O)CC(C3=O)(C)O)C(=C)C | yes | - 7.4 |
| 89 | Curcusone B | 175944 | CC1CC2=C(C1=O)C3C(C=C(C2=O)C)C(CCC3=C)C(=C)C | yes | - 7.1 |
| 90 | Curcusone D | 175945 | CC1=CC2C(CCC(=C)C2C3=C(C1=O)CC(C3=O)(C)O)C(=C)C | yes | - 7.1 |
| 91 | Inuviscolide | 176489 | CC1(CCC2C1CC3C(CC2=C)OC(=O)C3=C)O | Yes | - 7.2 |
| 92 | eurylene | 179304 | CC(=CCCC(C)(C1CCC(O1)(C)C(CCC(C2(CCC(O2)C(C)(CCC=C(C)C)O)C)OC(=O)C)OC(=O)C)O)C | Yes | - 7.4 |
| 93 | 23,24-dihydro-25-acetylcucurbitacin F | 181183 | CC(=O)OC(C)(C)CCC(=O)C(C)(C1C(CC2(C1(CC(=O)C3(C2CC=C4C3CC(C(C4(C)C)O)O)C)C)C)O)O | Yes | - 6.5 |
| 94 | Ergolide | 185786 | CC1CC2C(C(C3(C1CCC3=O)C)OC(=O)C)C(=C)C(=O)O2 | yes | - 6.2 |
| 95 | alpha-santonin | 221071 | CC1C2CCC3(C=CC(=O)C(=C3C2OC1=O)C)C | Yes | - 7.3 |
| 96 | Glaucarubol | 225484 | CC1C2C(C(=O)OC3C24COC(C1O)(C4C5(C(C3)C(=CC(C5O)O)C)C)O)O | yes | - 6.8 |
| 97 | Friedelin | 244297 | CC1C(=O)CCC2C1(CCC3C2(CCC4(C3(CCC5(C4CC(CC5)(C)C)C)C)C)C)C | yes | - 8.2 |
| 98 | Lupeol | 259846 | CC(=C)C1CCC2(C1C3CCC4C5(CCC(C(C5CCC4(C3(CC2)C)C)(C)C)O)C)C | yes | - 8.8 |
| 99 | Glaucarubolone | 277765 | CC1C2C(C(=O)OC3C24COC(C1O)(C4C5(C(C3)C(=CC(=O)C5O)C)C)O)O | yes | - 6.7 |
| 100 | Chaparrin | 280691 | CC1C2CC(=O)OC3C24COC(C1OC(=O)C)(C4C5(C(C3)C(=CC(C5OC(=O)C)OC(=O)C)C)C)O | yes | - 7.6 |
| 101 | acetylbetulinic acid | 289984 | CC(=C)C1CCC2(C1C3CCC4C5(CCC(C(C5CCC4(C3(CC2)C)C)(C)C)OC(=O)C)C)C(=O)O | Yes | - 7.4 |
| 102 | Jatropholone | 324064 | CC1CC2=C(C(=C3C4C(C4(C)C)CCC(=C)C3=C2C1=O)C)O | yes | - 7.7 |
| 103 | (-)-Perillylalcohol | 369312 | CC(=C)C1CCC(=CC1)CO | Yes | - 5.5 |
| 104 | Ilicic acid | 386212 | CC12CCCC(C1CC(CC2)C(=C)C(=O)O)(C)O | Yes | - 6.4 |
| 105 | Remangilones C | 397856 | CC1=C(C(=O)CC2(C1CCC3(C2CC=C4C3(C(C(=O)C5C4CC(CC5)(C)C)O)C)C)C)O | yes | - 8.7 |
| 106 | alpha-L-arabinopyranoside | 399180 | CC(CC(C1C(O1)(C)C)OC(=O)C)C2C(=O)C(C3(C2(CCC45C3CCC6C4(C5)CCC(C6(C)C)OC7C(C(C(CO7)O)O)O)C)C)O | Yes | - 8 |
| 107 | D-Limonene | 440917 | CC1=CCC(CC1)C(=C)C | yes | - 5.3 |
| 18 | Hyperforin | 441298 | CC(C)C(=O)C12C(=O)C(=C(C(C1=O)(CC(C2(C)CCC=C(C)C)CC=C(C)C)CC=C(C)C)O)CC=C(C)C | No |  |
| 109 | Cucurbitacin J | 441819 | CC1(C2=CCC3C4(CC(C(C4(CC(=O)C3(C2C=C(C1=O)O)C)C)C(C)(C(=O)CC(C(C)(C)O)O)O)O)C)C | yes | - 7.5 |
| 110 | Carnosol | 442009 | CC(C)C1=C(C(=C2C(=C1)C3CC4C2(CCCC4(C)C)C(=O)O3)O)O | yes | - 7.8 |
| 111 | (+)-Isopimaric acid | 442048 | CC1(CCC2C(=CCC3C2(CCCC3(C)C(=O)O)C)C1)C=C | Yes | - 7.2 |
| 112 | helenalin acetate | 442142 | CC1CC2C(C(C3(C1C=CC3=O)C)OC(=O)C)C(=C)C(=O)O2 | Yes | - 6.3 |
| 113 | canin | 442175 | CC1(CCC2C(C3C14C(O4)C5C3(O5)C)OC(=O)C2=C)O | Yes | - 6.8 |
| 114 | Michelenolide | 442278 | CC12CCC3C(C4C(O4)(CCC1O2)C)OC(=O)C3=C | Yes | - 7.1 |
| 115 | Alpha-Curcumene | 442360 | CC1=CC=C(C=C1)C(C)CCC=C(C)C | Yes | - 6.7 |
| 116 | Genipin | 442424 | COC(=O)C1=COC(C2C1CC=C2CO)O | yes | - 5.4 |
| 117 | Farnesol | 445070 | CC(=CCCC(=CCCC(=CCO)C)C)C | yes | - 6.3 |
| 118 | Lycopene | 446925 | CC(=CCCC(=CC=CC(=CC=CC(=CC=CC=C(C)C=CC=C(C)C=CC=C(C)CCC=C(C)C)C)C)C)C | No |  |
| 119 | Trans-Zeatin | 449093 | CC(=CCNC1=NC=NC2=C1NC=N2)CO | Yes | - 5.6 |
| 120 | Rosmanol | 455261 | CC(C)C1=C(C(=C2C(=C1)C(C3C4C2(CCCC4(C)C)C(=O)O3)O)O)O | yes | - 7.8 |
| 121 | 7-O-methylrosmanol | 455262 | CC(C)C1=C(C(=C2C(=C1)C(C3C4C2(CCCC4(C)C)C(=O)O3)OC)O)O | Yes | - 6.8 |
| 122 | longikaurin B | 457901 | CC(=O)OCC1(CCCC23C1C(C(C45C2CCC(C4O)C(=C)C5=O)(OC3)O)O)C | yes | - 7.2 |
| 123 | 3-oxo-friedelan-28 oic acid | 469744 | CC1C(=O)CCC2C1(CCC3C2(CCC4(C3(CCC5(C4CC(CC5)(C)C)C(=O)O)C)C)C)C | Yes | - 7.5 |
| 124 | Arnidiol | 470259 | CC1C2C3CCC4C5(CCC(C(C5CCC4(C3(CC(C2(CCC1=C)C)O)C)C)(C)C)O)C | yes | - 8.2 |
| 125 | alphitolic acid | 470606 | CC(=C)C1CCC2(C1C3=CCC4C(C3(CC2)C)(CCC5C4(CC(C(C5(C)C)O)O)C)C)C(=O)O | Yes | - 8.4 |
| 126 | 20-epibryonolic acid | 472768 | CC1(C2CCC3=C(C2(CCC1O)C)CCC4(C3(CCC5(C4CC(CC5)(C)C(=O)O)C)C)C)C | Yes | - 6.9 |
| 127 | Malacitanolide | 483221 | CC12CC(C3C(C1C(CCC2O)CO)OC(=O)C3=C)OC(=O)C(=C)C(CO)O | Yes | - 6.9 |
| 128 | Epi-betulinic acid | 485711 | CC(=C)C1CCC2(C1C3CCC4C5(CCC(C(C5CCC4(C3(CC2)C)C)(C)C)O)C)C(=O)O | yes | - 8.7 |
| 129 | 9-alpha-hydroxy-1-beta,10-alpha-epoxyparthenolide | 500219 | CC12CCC3C(O3)(C(CC4C(C1O2)OC(=O)C4=C)O)C | Yes | - 6.3 |
| 130 | alpha-cadinol | 519662 | CC1=CC2C(CCC(C2CC1)(C)O)C(C)C | Yes | - 6.4 |
| 131 | Spathulenol | 522266 | CC1(C2C1C3C(CCC3(C)O)C(=C)CC2)C | Yes | - 6.1 |
| 132 | 23,24-Dihydrocucurbitacin B | 588303 | CC(=O)OC(C)(C)CCC(=O)C(C)(C1C(CC2(C1(CC(=O)C3(C2CC=C4C3CC(C(=O)C4(C)C)O)C)C)C)O)O | Yes | - 6.8 |
| 133 | Canophyllic acid | 596679 | CC1C(CCC2C1(CCC3C2(CCC4(C3(CCC5(C4CC(CC5)(C)C)C(=O)O)C)C)C)C)O | yes | - 8.4ss |
| 134 | 7-hydroxycadallin | 608115 | CC1=C2C=C(C(=CC2=C(C=C1)C(C)C)C)O | Yes | - 6.2 |
| 135 | 2-alpha-hydroxytirotundin | 636756 | CC1CC2C(C(CC3(CC(C1(O3)O)O)C)OC(=O)C(C)C)C(=C)C(=O)O2 | Yes | - 6.7 |
| 136 | Taxumairol Q | 637469 | CC1=C2C(C(C3(C(CC4C(C3C(C2(CC1O)C(C)(C)O)OC(=O)C)(CO4)OC(=O)C)O)C)O)O | Yes | - 5.8 |
| 137 | Geraniol | 637566 | CC(=CCCC(=CCO)C)C | yes | - 5.2 |
| 138 | Citral | 638011 | CC(=CCCC(=CC=O)C)C | Yes | - 5.5 |
| 139 | Beta-Ionone | 638014 | CC1=C(C(CCC1)(C)C)C=CC(=O)C | yes | - 6.1 |
| 140 | Squalene | 638072 | CC(=CCCC(=CCCC(=CCCC=C(C)CCC=C(C)CCC=C(C)C)C)C)C | yes | - 6.6 |
| 141 | (23E)-coumaroylhederagenin | 643707 | CC1(CCC2(CCC3(C(=CCC4C3(CCC5C4(CCC(C5(C)COC(=O)C=CC6=CC=C(C=C6)O)O)C)C)C2C1)C)C(=O)O)C | No | - 7.9 |
| 142 | Vibsanins P | 643712 | CC(=CC(=O)OC1C=CC(CC=C(CCC2C1(O2)C)CO)(C)CC=CC(C)(C)O)C | Yes | - 7.1 |
| 143 | Nerol | 643820 | CC(=CCCC(=CCO)C)C | yes | - 4.9 |
| 144 | Neryl acetate | 1549025 | CC(=CCCC(=CCOC(=O)C)C)C | yes | - 5.6 |
| 145 | Geranyl acetate | 1549026 | CC(=CCCC(=CCOC(=O)C)C)C | yes | - 6 |
| 146 | Geranyl acetone | 1549778 | CC(=CCC/C(=C/CCC(=O)C)/C)C | yes | - 6.1 |
| 147 | beta-caryophyllene oxide | 1742210 | CC1(CC2C1CCC3(C(O3)CCC2=C)C)C | Yes | - 6.2 |
| 148 | Terpinen-4-ol | 2724161 | CC1=CCC(CC1)(C(C)C)O | yes | - 5.2 |
| 149 | Bisabolol | 3033866 | CC1=CCC(=C(C)CCC=C(C)C)CC1 | yes | - 6.8 |
| 150 | 12-Deoxyphorbol 20-acetate 13-phenylacetate | 3034821 | CC1CC2(C(C2(C)C)C3C1(C4C=C(C(=O)C4(CC(=C3)COC(=O)C)O)C)O)OC(=O)CC5=CC=CC=C5 | Yes | - 7.9 |
| 151 | eurycomanone | 3069398 | CC1=CC(=O)C(C2(C1CC3C45C2C(C(C(=C)C4(C(C(=O)O3)O)O)O)(OC5)O)C)O | Yes | - 7.3 |
| 152 | Bigelovin | 3080597 | CC1CC2C(C(C3(C1C=CC3=O)C)OC(=O)C)C(=C)C(=O)O2 | yes | - 6.8 |
| 153 | T-muurolol | 3084331 | CC1=CC2C(CCC(C2CC1)(C)O)C(C)C | Yes | - 6.4 |
| 154 | Ent-8,9-Seco-7-alpha,11-beta-dihydroxykaura-8(14),16-dien-9,15-dione | 3809845 | CC1(CCCC2(C1CC(C3=CC(CC(C2=O)O)C(=C)C3=O)O)C)C | yes | - 6.6 |
| 155 | Uncarinic acid A | 5274626 | CC1(CCC2(CCC3(C(=CCC4C3(CCC5C4(CCC(C5(C)C)O)C)C)C2C1)C(=O)C=CC6=CC(=C(C=C6)O)O)C(=O)O)C | No |  |
| 156 | Geranic acid | 5275520 | CC(=CCCC(=CC(=O)O)C)C | yes | - 5.5 |
| 157 | Zeaxanthin | 5280899 | CC1=C(C(CC(C1)O)(C)C)C=CC(=CC=CC(=CC=CC=C(C)C=CC=C(C)C=CC2=C(CC(CC2(C)C)O)C)C)C | No |  |
| 158 | Crocetin | 5281232 | CC(=CC=CC=C(C)C=CC=C(C)C(=O)O)C=CC=C(C)C(=O)O | yes | - 6.3 |
| 159 | Crocin | 5281233 | CC(=CC=CC=C(C)C=CC=C(C)C(=O)OC1C(C(C(C(O1)COC2C(C(C(C(O2)CO)O)O)O)O)O)O)C=CC=C(C)C(=O)OC3C(C(C(C(O3)COC4C(C(C(C(O4)CO)O)O)O)O)O)O | no |  |
| 160 | Lutein | 5281243 | CC1=C(C(CC(C1)O)(C)C)C=CC(=CC=CC(=CC=CC=C(C)C=CC=C(C)C=CC2C(=CC(CC2(C)C)O)C)C)C | No |  |
| 161 | Neoxanthin | 5281247 | CC(=CC=CC=C(C)C=CC=C(C)C=C=C1C(CC(CC1(C)O)O)(C)C)C=CC=C(C)C=CC23C(CC(CC2(O3)C)O)(C)C | No |  |
| 162 | Cucurbitacin A | 5281315 | CC(=O)OC(C)(C)C=CC(=O)C(C)(C1C(CC2(C1(CC(=O)C3(C2CC=C4C3CC(C(=O)C4(C)C)O)CO)C)C)O)O | yes | - 6.9 |
| 163 | Cucurbitacin B | 5281316 | CC(=O)OC(C)(C)C=CC(=O)C(C)(C1C(CC2(C1(CC(=O)C3(C2CC=C4C3CC(C(=O)C4(C)C)O)C)C)C)O)O | yes | - 7.3 |
| 164 | Cucurbitacin D | 5281318 | CC1(C2=CCC3C4(CC(C(C4(CC(=O)C3(C2CC(C1=O)O)C)C)C(C)(C(=O)C=CC(C)(C)O)O)O)C)C | yes | - 7.1 |
| 165 | Cucurbitacin I | 5281321 | CC1(C2=CCC3C4(CC(C(C4(CC(=O)C3(C2C=C(C1=O)O)C)C)C(C)(C(=O)C=CC(C)(C)O)O)O)C)C | yes | - 7 |
| 166 | Cucurbitacin Q | 5281323 | CC(=O)OC(C)(C)C=CC(=O)C(C)(C1C(CC2(C1(CC(=O)C3(C2CC=C4C3CC(C(C4(C)C)O)O)C)C)C)O)O | yes | - 6.5 |
| 167 | Costunolide | 5281437 | CC1=CCCC(=CC2C(CC1)C(=C)C(=O)O2)C | yes | - 6.6 |
| 168 | Xanthatin | 5281511 | CC1CC2C(CC=C1C=CC(=O)C)C(=C)C(=O)O2 | Yes | - 6.6 |
| 169 | Beta-Caryophyllene | 5281515 | CC1=CCCC(=C)C2CC(C2CC1)(C)C | yes | - 6 |
| 170 | Beta-Farnesene | 5281517 | CC(=CCCC(=CCCC(=C)C=C)C)C | yes | - 6.1 |
| 171 | Alpha-Humulene | 5281520 | CC1=CCC(C=CCC(=CCC1)C)(C)C | Yes | - 6 |
| 172 | Ocimene | 5281553 | CC(=CCC=C(C)C=C)C | yes | - 5.6 |
| 173 | Alpha-Ionone | 5282108 | CC1=CCCC(C1C=CC(=O)C)(C)C | Yes | - 5.6 |
| 174 | D-alpha-tocotrienol | 5282347 | CC1=C(C2=C(CCC(O2)(C)CCC=C(C)CCC=C(C)CCC=C(C)C)C(=C1O)C)C | yes | - 7.4 |
| 175 | D-gamma-tocotrienol | 5282349 | CC1=C(C=C2CCC(OC2=C1C)(C)CCC=C(C)CCC=C(C)CCC=C(C)C)O | yes | - 7.5 |
| 176 | D-delta-tocotrienol | 5282350 | CC1=CC(=CC2=C1OC(CC2)(C)CCC=C(C)CCC=C(C)CCC=C(C)C)O | yes | - 7.2 |
| 177 | Nerolidol | 5284507 | CC(=CCCC(=CCCC(C)(C=C)O)C)C | yes | - 6.1 |
| 178 | 3-o-(cis-p-coumaroyl)-alphitolic acid | 5316118 | CC(=C)C1CCC2(C1C3(CCC4C(C3(CC2)C)(CCC5C4(CC(C(C5(C)C)OC(=O)C=CC6=CC=C(C=C6)O)O)C)C)C)C(=O)O | No |  |
| 179 | 3-o-(cis-p-coumaroyl)-maslinic acid | 5316120 | CC1(CCC2(CCC3(C(=CCC4C3(CCC5C4(CC(C(C5(C)C)OC(=O)C=CC6=CC=C(C=C6)O)O)C)C)C2C1)C)C(=O)O)C | No |  |
| 180 | 1-deoxy-3-methacrylyl-11-methoxymeliacarpinin | 5316567 | CC(=C)C(=O)OC1CCC23COC(C2C4(C(C5C3C1(CO5)C)OC6(C4(C7CC6C8(C=COC8O7)O)O)C)C)(C(=O)OC)OC | No |  |
| 181 | 2-alpha-hydroxyursolic acid | 5318379 | CC1CCC2(CCC3(C(=CCC4C3(CCC5C4(CC(C(C5(C)C)O)O)C)C)C2C1C)C)C(=O)O | Yes | - 7.7 |
| 182 | Andrographolide | 5318517 | CC12CCC(C(C1CCC(=C)C2CC=C3C(COC3=O)O)(C)CO)O | Yes | - 6.8 |
| 183 | 21-O-methyltoosendanpentol | 5319791 | CC1(C(CCC2(C1CC(C3(C2CCC4(C3=CCC4C5CC(OC5OC)C(C(C)(C)O)O)C)C)O)C)O)C | Yes | - 7.4 |
| 184 | Pulsatillic acid | 5320798 | CC(=C)C1CCC2(C1C3CCC4C5(CCC(=O)C(C5CCC4(C3(CC2)C)C)(C)CO)C)C(=O)O | yes | - 7.3 |
| 185 | Sulphurenic acid | 5321559 | CC(C)C(=C)CCC(C1CC(C2(C1(CCC3=C2CCC4C3(CCC(C4(C)C)O)C)C)C)O)C(=O)O | Yes | - 8.4 |
| 186 | taxuspine | 5321748 | CC1=C2C(C(C3(C(CC(C(=C)C3C(C(C2(C)C)CC1=O)OC(=O)C)O)OC(=O)C)C)OC(=O)C)OC(=O)C | Yes | - 6.1 |
| 187 | gamma-caryophyllene | 5322111 | CC1=CCCC(=C)C2CC(C2CC1)(C)C | Yes | - 6.6 |
| 188 | Buddledin C | 5352042 | CC1=CCCC(=C)C2CC(C2CC1=O)(C)C | yes | - 6.2 |
| 189 | Casearborin A | 5352094 | CC1CCC23C(C1(C)CC=C(C)C=C)CC(C=C2C(OC3OC(=O)C)OC(=O)C)OC(=O)C4=CC=C(C=C4)O | yes | - 7.2 |
| 190 | Casearborin B | 5352095 | CC1CCC23C(C1(C)CC=C(C)C=C)CC(C=C2C(OC3OC(=O)C)OC(=O)C)OC(=O)C4=CC(=C(C=C4)O)OC | yes | - 7.5 |
| 191 | Cucurbitacin E | 5353547 | CC(=O)OC(C)(C)C=CC(=O)C(C)(C1C(CC2(C1(CC(=O)C3(C2CC=C4C3C=C(C(=O)C4(C)C)O)C)C)C)O)O | yes | - 7.5 |
| 192 | Geranyl butyrate | 5355856 | CCCC(=O)OCC=C(C)CCC=C(C)C | yes | - 6 |
| 193 | Tagitinin C | 5358948 | CC1=CC2C(C(CC(C=CC1=O)(C)O)OC(=O)C(C)C)C(=C)C(=O)O2 | yes | - 6.7 |
| 194 | Argentinic acid D methyl ester | 5471744 | CCC(C)C(C(=O)OC1CC2(C(CC=C2C3(C1C(C(CC3OC(=O)C(C(=CC)C)O)C(C)(C)O)(C)CCC(=O)OC)C)C4CC(OC4)C=C(C)C)C)O | Yes |  |
| 195 | Cucurbitacin F | 5476663 | CC1(C(C(CC2C1=CCC3C2(C(=O)CC4(C3(CC(C4C(C)(C(=O)C=CC(C)(C)O)O)O)C)C)C)O)O)C | yes | - 7.4 |
| 196 | Buddledin A | 5477391 | CC1=CCCC(=C)C2CC(C2C(C1=O)OC(=O)C)(C)C | yes | - 6.1 |
| 197 | 20-hydroxyresiniferol 9,13,-14-orthophenylacetate | 5490349 | CC1CC2(C3C4C1(C5C=C(C(=O)C5(CC(=C4)CO)O)C)OC(O3)(O2)CC6=CC=CC=C6)C(=C)C | Yes | - 8.2 |
| 198 | Casearborin C | 6367397 | CC1CC(C23C(C1(C)CC=C(C)C=C)CC(C=C2C(OC3OC(=O)C)OC(=O)C)O)OC(=O)C4=CC=C(C=C4)O | yes | - 7.8 |
| 199 | Casearborin D | 6367398 | CC1CC(C23C(C1(C)CC=C(C)C=C)CC(C=C2C(OC3OC(=O)C)OC(=O)C)OC(=O)C4=CC=C(C=C4)O)O | yes | - 7.9 |
| 200 | Casearborin E | 6367399 | CC1CC(C23C(C1(C)CC=C(C)C=C)CC(C=C2C(OC3OC(=O)C)OC(=O)C)OC(=O)C)OC(=O)C4=CC=C(C=C4)O | yes | - 7.8 |
| 201 | Ent-8,9-Seco-7-alpha-acetoxykaura-8(14),16-dien-9,15-dione | 6398570 | CC(=O)OC1CC2C(CCCC2(C(=O)CCC3C=C1C(=O)C3=C)C)(C)C | yes | - 6.6 |
| 202 | Ent-8,9-Seco-7-alpha-hydroxy-11-acetoxykaura-8(14),16-dien-9,15-dione | 6398572 | CC(=O)OC1CC2C=C(C(CC3C(CCCC3(C1=O)C)(C)C)O)C(=O)C2=C | Yes | - 7 |
| 203 | Bisabolone | 6429340 | CC1=CCC(CC1)C(C)CCC(=O)C(C)C | yes | - 6.3 |
| 204 | Bruceantin | 6434323 | CC1=C(C(=O)CC2(C1CC3C45C2C(C(C(C4C(C(=O)O3)OC(=O)C=C(C)C(C)C)(OC5)C(=O)OC)O)O)C)O | no |  |
| 205 | Lantadene A | 6436598 | CC=C(C)C(=O)OC1CC(CC2C1(CCC3(C2=CCC4C3(CCC5C4(CCC(=O)C5(C)C)C)C)C)C(=O)O)(C)C | No |  |
| 206 | Trichothecin | 6436600 | CC=CC(=O)OC1CC2C3(C1(C4(CC(=O)C(=CC4O2)C)C)C)CO3 | Yes | - 7.1 |
| 207 | ridentin | 6441492 | CC1=CC2C(CCC(=C)C(CC1O)O)C(=C)C(=O)O2 | Yes | - 6.6 |
| 208 | 12-Deoxyphorbol 20-acetate 13-angelate | 6444377 | CC=C(C)C(=O)OC12CC(C3(C(C1C2(C)C)C=C(CC4(C3C=C(C4=O)C)O)COC(=O)C)O)C | Yes | - 7.1 |
| 209 | Parthenolide | 6473881 | CC1=CCCC2(C(O2)C3C(CC1)C(=C)C(=O)O3)C | yes | - 6.3 |
| 210 | Cnicin | 6475844 | CC1=CCCC(=CC2C(C(C1)OC(=O)C(=C)C(CO)O)C(=C)C(=O)O2)CO | yes | - 6.7 |
| 211 | 3-beta-O-(E)-coumaroylbetulin | 6479753 | CC(=C)C1CCC2(C1C3CCC4C5(CCC(C(C5CCC4(C3(CC2)C)C)(C)C)OC(=O)C=CC6=CC=C(C=C6)O)C)C(=O)O | No |  |
| 212 | 20-hydroxy-12-deoxyphorbol angelate | 6506231 | CC=C(C)C(=O)OC12CC(C3(C(C1C2(C)C)C=C(CC4(C3C=C(C4=O)C)O)CO)O)C | Yes | - 7 |
| 213 | 27-deoxyactein | 6537501 | CC1CC2(C3C(O3)(CO2)C)OC4C1C5(CCC67CC68CCC(C(C8CCC7C5(C4)C)(C)C)OC9C(C(C(CO9)O)O)O)C | Yes | - 8.6 |
| 214 | Olean-12-en-3-beta-ol | 6708529 | CC1(CCC2(CCC3(C(=CCC4C3(CCC5C4(CCC(C5(C)C)O)C)C)C2C1)C)C)C | yes | - 8.5 |
| 215 | Simalikalactone D | 6711208 | CCC(C)C(=O)OC1C2C3(C(C(C4C2(CO3)C(CC5C4(C(C(=O)C=C5C)O)C)OC1=O)O)O)C | yes | - 6.9 |
| 216 | Corosolic acid | 6918774 | CC1CCC2(CCC3(C(=CCC4C3(CCC5C4(CC(C(C5(C)C)O)O)C)C)C2C1C)C)C(=O)O | yes | - 8.1 |
| 217 | betulonic acid | 9933683 | CC(=C)C1CCC2(C1C3CCC4C5(CCC(=O)C(C5CCC4(C3(CC2)C)C)(C)C)C)C(=O)O | Yes | - 8.2 |
| 218 | 7-epi-7-O-methylrosmanol | 9950773 | CC(C)C1=C(C(=C2C(=C1)C(C3C4C2(CCCC4(C)C)C(=O)O3)OC)O)O | Yes | - 8 |
| 219 | 12-O-Methylcarnosicacid | 9974918 | CC(C)C1=C(C(=C2C(=C1)CCC3C2(CCCC3(C)C)C(=O)O)O)OC | Yes | - 7.2 |
| 220 | 3-beta-acetoxy-8-beta-isobutyryloxyreynosin | 9977821 | CC(C)C(=O)OC1CC2(C(CC(C(=C)C2C3C1C(=C)C(=O)O3)OC(=O)C)O)C | Yes | - 7.1 |
| 221 | 3,6-Epidioxy-1,10-bisaboladiene | 10014355 | CC(CCC=C(C)C)C12CCC(C=C1)(OO2)C | Yes | - 6.3 |
| 222 | Wikstroelides J | 10032323 | CCCCCCCCCC=CC=CC(=O)OC1C2C3C(O3)(C(C4(C(C2(C(C(C1(C(=C)C)O)OC(=O)C)C)O)C=C(C4=O)C)O)O)CO | No |  |
| 223 | Trichilin H | 10032674 | CC(C)C(=O)OC1C2(C3CC(C4(C(C3(CO1)C(C(C2OC(=O)C)OC(=O)C)O)C(=O)C(C5(C46C(O6)CC5C7=COC=C7)C)OC(=O)C)C)O)C | No |  |
| 224 | inuloidin | 10059834 | CC1=CC2=C(C=C1O)C(=C)C(CC2C(C)C)O | yes | - 6.8 |
| 225 | 12-Hydroxychiloscyphone | 10243131 | CC1CCC=C2C1(C(CC2)C(=O)C(=C)CO)C | Yes | - 6.3 |
| 226 | Ferruginin A | 10253728 | CC1=CC2=CC3=C(C(=CC(=O)C3(CC=C(C)C)CC=C(C)C)O)C(=C2C(=C1CC=C(C)C)O)O | Yes | - 8 |
| 227 | Reissantins E | 10323282 | CC(=O)OC1CCC(C23C1(C(C(C(C2O)C(O3)(C)C)OC(=O)C)OC(=O)C4=CC=CC=C4)C)(C)O | yes | - 6.6 |
| 228 | 1-cinnamoyl-3-acetyl-11-methoxymeliacarpinin | 10327653 | CC(=O)OC1CC(C23COC(C2C4(C(C5C3C1(CO5)C)OC6(C4(C7CC6C8(C=COC8O7)O)O)C)C)(C(=O)OC)OC)OC(=O)C=CC9=CC=CC=C9 | No |  |
| 229 | Ent-Kaur-16-en-15-one | 10334208 | CC1(CCCC2(C1CCC34C2CCC(C3)C(=C)C4=O)C)C | Yes | - 7.9 |
| 230 | Melissoidesin G | 10342874 | CC(=O)OC1CCC2(C3C(CC4CC3(C(C(C2C1(C)C)OC(=O)C)O)C(=O)C4=C)O)C | Yes | - 6.5 |
| 231 | Reissantins D | 10343634 | CC(=O)OC1CCC(C23C1(C(C(C(C2O)C(O3)(C)C)O)OC(=O)C4=CC=CC=C4)C)(C)O | yes | - 6.8 |
| 232 | 12-Deoxyphorbol 13-(9,10-methylene)undecanoate | 10369667 | CC1CC1CCCCCCCC(=O)OC23CC(C4(C(C2C3(C)C)C=C(CC5(C4C=C(C5=O)C)O)CO)O)C | Yes | - 6.5 |
| 233 | 17-(methoxycarbonyl)-28-nor-isoiguesterin | 10411393 | CC1=C(C(=O)C=C2C1=CC=C3C2(CCC4(C3(CCC5(C4CC(=C)CC5)C(=O)OC)C)C)C)O | Yes | - 7.6 |
| 234 | Vibsanins W | 10411484 | CC(=CC(=O)OC=CC1C(C(=O)C(=CCC1(C)CCC(C(C)(C)O)O)CO)CC(=O)C)C | Yes | - 6.7 |
| 235 | Dawoensin A | 10412708 | CC(=O)OC1CCC2(C3C(CC4CC3(C(C(C2C1(C)C)OC(=O)C)OC(=O)C)C(=O)C4=C)O)C | Yes | - 6.3 |
| 236 | Bucidarasin B | 10414276 | CCC(C)C(=O)OC1CC2C(C(CC(C23C(OC(C3=C1)OC(=O)C)OC(=O)C)O)C)(C)CC=C(C)C=C | yes | - 6.6 |
| 237 | 4-alpha,6-alpha-dihydroxyeudesman-8-beta,12-olide | 10445633 | CC12CCCC(C1C(C3C(C2)OC(=O)C3=C)O)(C)O | Yes | - 6.4 |
| 238 | 8-dihydrotrichothecinol A | 10449549 | CC=CC(=O)OC1C(C2C3(C1(C4(CCC(=CC4O2)C)C)C)CO3)O | Yes | - 6.7 |
| 239 | Cedronolactones B | 10451401 | CC1C2CC(=O)OC3C24COC(C1O)(C4C(C3)(C)C5C(=CC(=O)O5)C)O | yes | - 7.1 |
| 240 | 1-beta,6-alpha-dihydroxy-4(15)-eudesmene | 10466743 | CC(C)C1CCC2(C(CCC(=C)C2C1O)O)C | Yes | - 5.9 |
| 241 | Trichothecinol A | 10472929 | CC=CC(=O)OC1C(C2C3(C1(C4(CC(=O)C(=CC4O2)C)C)C)CO3)O | Yes | - 7.4 |
| 242 | Isoiguesterol | 10477355 | CC1=C(C(=O)C=C2C1=CC=C3C2(CCC4(C3(CCC5(C4CC(CC5)CO)C)C)C)C)O | Yes | - 8.3 |
| 243 | Reissantins B | 10485185 | CC(=O)OC1C2C(C3(C(CC(C(C3(C1OC(=O)C4=CC=CC=C4)C)OC(=O)C)OC(=O)C5=CC=CC=C5)(C)O)OC2(C)C)OC(=O)C6=CN(C(=O)C=C6)C | No |  |
| 244 | Remangilones A | 10503181 | CC1=C2CCC3(C(C2(C=C(C1=O)O)C)CC=C4C3(C(C(=O)C5C4CC(CC5)(C)C)O)C)C | yes | - 7.4 |
| 245 | 1-cinnamoyl-3-hydroxy-11-methoxymeliacarpinin | 10508889 | CC12COC3C1C4(COC(C4C5(C3OC6(C5(C7CC6C8(C=COC8O7)O)O)C)C)(C(=O)OC)OC)C(CC2O)OC(=O)C=CC9=CC=CC=C9 | No |  |
| 246 | Cedronolactones D | 10548655 | CC1=CC(=O)C(C2(C1CC(C34C2C(C5C(C3C(C(=O)O5)O)(OC4)C)O)O)C)O | yes | - 7.1 |
| 247 | 5-epi-vibsanin H | 10550692 | CC(=CC(=O)OC=CC1C(C(=O)C(=CCC1(C)CC=CC(C)(C)O)CO)CC(=O)C)C | Yes | - 6.8 |
| 248 | Zhankuic acid A | 10600386 | CC1C2CC(=O)C3=C(C2(CCC1=O)C)C(=O)CC4(C3CCC4C(C)CCC(=C)C(C)C(=O)O)C | Yes | - 8.2 |
| 249 | Ent-14-beta-Hydroxykaur-16-en-15-one | 10614344 | CC1(CCCC2(C1CCC34C2CCC(C3O)C(=C)C4=O)C)C | yes | - 7.4 |
| 250 | 3-beta-acetoxy-25-hydroxylanosta-8,23-diene | 10624741 | CC(CC=CC(C)(C)O)C1CCC2(C1(CCC3C2=CCC4C3(CCC(C4(C)C)OC(=O)C)C)C)C | Yes | - 7 |
| 251 | Ent-8,9-Seco-7-alpha-hydroxy-17-thiophenylkaur-8(14)-en-9,15-dione | 10670128 | CC1(CCCC2(C1CC(C3=CC(CCC2=O)C(C3=O)CSC4=CC=CC=C4)O)C)C | yes | - 7.6 |
| 252 | Ent-7-alpha-Acetoxy-14-beta-hydroxykaur-16-en-15-one | 10713565 | CC(=O)OC1CC2C(CCCC2(C3C14C(C(CC3)C(=C)C4=O)O)C)(C)C | yes | - 7.1 |
| 253 | 3-beta-Hydroxy-27-p-(Z)-coumaroyloxyolean-12-en-28-oic acid | 10746421 | CC1(CCC2(CCC3(C(=CCC4C3(CCC5C4(CCC(C5(C)C)O)C)C)C2C1)COC(=O)C=CC6=CC=C(C=C6)O)C(=O)O)C | No |  |
| 254 | Ent-8,9-Seco-7-alpha-acetoxy-11-beta-hydroxykaura-8(14),16-dien-9,15-dione | 10785623 | CC(=O)OC1CC2C(CCCC2(C(=O)C(CC3C=C1C(=O)C3=C)O)C)(C)C | Yes | - 6.7 |
| 255 | 12-Acetoxy-5-hydroxynerolidol | 10827791 | CC(=CC(CC(C)(C=C)O)O)CCC=C(C)COC(=O)C | Yes | - 5.8 |
| 256 | Zhankuic acid C | 10838646 | CC1C(CCC2(C1CC(=O)C3=C2C(=O)C(C4(C3CCC4C(C)CCC(=C)C(C)C(=O)O)C)O)C)O | Yes | - 7.8 |
| 257 | Adenanthin F | 10873754 | CC(=O)OC1CC2C(C(CC(C2(C3C14CC(CC3O)C(=C)C4=O)C)O)OC(=O)C)(C)C | Yes | - 7.4 |
| 258 | 14,15-beta-dihydroxyklaineanone | 10883790 | CC1C(C(C2C3(C(CC4C2(C1(C(C(=O)O4)O)O)C)C(=CC(=O)C3O)C)C)O)O | Yes | - 6.9 |
| 259 | Adenanthin C | 10928875 | CC(=O)OC1CC2CC3(C1C4(C(CC3OC(=O)C)C(C(CC4OC(=O)C)O)(C)C)C)C(=O)C2=C | Yes | - 7.1 |
| 260 | Tithofolinolide | 10938482 | CC(C)C(=O)OC1CC2(C(CC(C(C2C3C1C(=C)C(=O)O3)(C)O)OC(=O)C)O)C | yes | - 7.3 |
| 261 | 3-alpha-acetoxydiversifolol | 11120895 | CC(=O)OC1CCC2(CCC(CC2C1(C)O)C=CC(=O)OC)C | Yes | - 6.9 |
| 262 | Vibsanins Q | 11154724 | CC(=CC(=O)OC1C=CC(CC=C(CCC2C1(O2)C)CO)(C)CC=CC(C)(C)OC)C | Yes | - 7.5 |
| 263 | Melissoidesins N | 11164768 | CC(=O)OC1CC23CC(CC(C2C4(C1C(C(CC4)O)(C)C)C)O)C(=C)C3=O | Yes | - 6.8 |
| 264 | Javanicoside C | 11169890 | CC1=C2CC3C45COC(C4C(C(=O)O3)OC(=O)C=C(C)C)(C(C(C5C2(C=C(C1=O)OC6C(C(C(C(O6)CO)O)O)O)C)O)O)C(=O)OC | No |  |
| 265 | Javanicoside B | 11169907 | CC1C=C(C(=O)C2(C1CC3C45C2C(C(C(C4C(C(=O)O3)OC(=O)CC(C)C)(OC5)C(=O)OC)O)O)C)OC6C(C(C(C(O6)CO)O)O)O | No |  |
| 266 | Taiwaniaquinol D | 11175350 | CC(C)C1=C(C2=C(C(=C1OC)O)C3(CCCC(C3=C2C=O)(C)C)C)O | Yes | - 7 |
| 267 | Javanicolides C | 11214625 | CC1C2CC3C45COC(C4C(C(=O)O3)OC(=O)C=C(C)C)(C(C(C5C2(CC(C1O)O)C)O)O)C(=O)OC | No |  |
| 268 | Javanicoside D | 11227971 | CCC(=CC(=O)OC1C2C34COC2(C(C(C3C5(C=C(C(=O)C(C5CC4OC1=O)C)OC6C(C(C(C(O6)CO)O)O)O)C)O)O)C(=O)OC)C(C)(C)O | No |  |
| 269 | Vibsanins R | 11258097 | CC(=CC(=O)OC1C=CC(CC=C(CCC2C1(O2)C)CO)(C)CC=CC(=C)C)C | Yes | - 7.1 |
| 270 | Vibsanins V | 11267134 | CC(=C)C(CCC1(CC=C(CCC2C(O2)(CC=C1)C)C=O)C)O | Yes | - 6.7 |
| 271 | Javanicoside E | 11274270 | CC1C2CC3C45COC(C4C(C(=O)O3)OC(=O)CC(C)C(C)(C)OC(=O)C)(C(C(C5C2(C=C(C1=O)OC6C(C(C(C(O6)CO)O)O)O)C)O)O)C(=O)OC | No |  |
| 272 | Taiwaniaquinone H | 11290884 | CC(C)C1=C(C(=O)C2=C(C1=O)C(C3C2(CCCC3(C)C)C)C=O)OC | Yes | - 7.1 |
| 273 | Taiwaniaquinone F | 11290884 | CC(C)C1=C(C(=O)C2=C(C1=O)C(C3C2(CCCC3(C)C)C)C=O)OC | Yes | - 7.1 |
| 274 | Vibsanins S | 11293010 | CC(=CC(=O)OC1C=CC(CC=C(CCC2C1(O2)C)CO)(C)CCC(C(=C)C)O)C | Yes | - 6.7 |
| 275 | 5-epi-Vibsanin C | 11327504 | CC(=CCCC1(CC=C(C(=O)C(C1C=COC(=O)C=C(C)C)CC(=O)C)CO)C)C | Yes | - 7.1 |
| 276 | Mitrekaurenone | 11381465 | CC12CCCC3(C1C(C(=O)C45C2CCC(C4)C(=C)C5)OC3=O)C | Yes | - 7.7 |
| 277 | Vibsanins T | 11441439 | CC(=CC(=O)OC1C=CC(CC=C(CCC2C1(O2)C)C=O)(C)CC=CC(C)(C)O)C | Yes | - 7.4 |
| 278 | Javanicoside F | 11445415 | CCC(=CC(=O)OC1C2C34COC2(C(C(C3C5(C=C(C(=O)C(C5CC4OC1=O)C)OC6C(C(C(C(O6)CO)O)O)O)C)O)O)C(=O)OC)C | No |  |
| 279 | 4-alpha,10-alpha-dihydroxy-3-oxo-8-beta-isobutyryloxyguaia-11(13)-en-12,6-alpha-olide | 11474040 | CC(C)C(=O)OC1CC(C2CC(=O)C(C2C3C1C(=C)C(=O)O3)(C)O)(C)O | Yes | - 7.4 |
| 280 | 1-(7-hydroxy-2,6-dimethyl-1-naphthyl)-4-methyl-3-pentanone | 11529140 | CC1=C(C2=C(C=C1)C=C(C(=C2)O)C)CCC(=O)C(C)C | Yes | - 6.7 |
| 281 | 3-beta ,12-dihydroxy-13-methyl-6,8,11,13-podocarpatetraen | 11644754 | CC1=CC2=C(C=C1O)C3(CCC(C(C3C=C2)(C)C)O)C | Yes | - 7.8 |
| 282 | Taiwaniaquinol B | 11688549 | CC(C)C1=C(C2=C(C(=C1OC)O)C3(CCCC(C3C2=O)(C)C)C)O | Yes | - 6.6 |
| 283 | 3beta ,12-dihydroxy-13-methyl-5,8,11,13-podocarpatetraen-7-one | 11694896 | CC1=CC2=C(C=C1O)C3(CCC(C(C3=CC2=O)(C)C)O)C | Yes | - 7.9 |
| 284 | Clerodane diterpenoid | 11723436 | CC1CCC2(C(C1(C)CCC3=CC(=O)OC3O)CCC=C2C)C | yes | - 7.6 |
| 285 | Cedronolactones A | 11754814 | CC1=CC(=O)C(C2(C1CC3C45C2C(C(C(C4C(C(=O)O3)OC(=O)CC(C)C)(OC5)C)O)O)C)O | yes | - 7.1 |
| 286 | Bucidarasin C | 11755249 | CC1CCC23C(C1(C)CC=C(C)C=C)CC(C=C2C(OC3OC(=O)C)OC(=O)C)OC(=O)C(C)C | yes | - 6.4 |
| 287 | Reissantins A | 11763527 | CC(=O)OC1CCC(C23C1(C(C(C(C2OC(=O)C4=CN(C(=O)C=C4)C)C(O3)(C)C)OC(=O)C)OC(=O)C5=CC=CC=C5)C)(C)O | No |  |
| 288 | Silvestrol | 11787114 | COC1C(OC(CO1)C(CO)O)OC2=CC3=C(C(=C2)OC)C4(C(C(C(C4(O3)C5=CC=C(C=C5)OC)C6=CC=CC=C6)C(=O)OC)O)O | No |  |
| 289 | Maytenfolone-A | 11798592 | CC1C(=O)CCC2C1(CCC3C2(CCC4(C3(CC(C56C4CC(CC5)(C(=O)OC6)C)O)C)C)C)C | yes | - 8.2 |
| 290 | Allobetulin | 11954143 | CC1(CCC23CCC4(C(C2C1OC3)CCC5C4(CCC6C5(CCC(C6(C)C)O)C)C)C)C | Yes | - 7.9 |
| 291 | Taiwaniaquinone D | 11962188 | CC(C)C1=C(C2=C(C(=O)C1=O)C3(CCCC(C3=C2C=O)(C)C)C)O | Yes | - 7.4 |
| 292 | Escobarine A | 11983334 | CC1(CCCC2(C1CC(C3C2CC(=O)C4(C3(O4)C=O)C#C)O)C)C | yes | - 7.3 |
| 293 | Escobarine B | 11983335 | CC1(CCCC2(C1CC(C3C2CC(=O)C4(C3(O4)CO)C#C)O)C)C | yes | - 7.1 |
| 294 | 11-beta,13-dihydroparthenolide | 12038773 | CC1=C2C(C(C3(C(CC4C(C3C(C2(CC1OC(=O)C5=CC=CC=C5)C(C)(C)O)OC(=O)C)(CO4)OC(=O)C)OC(=O)C)C)OC(=O)C)OC(=O)C6=CC=CC=C6 | No |  |
| 295 | 28-oxoallobetulin | 12305935 | CC1(CCC23CCC4(C(C2C1OC3=O)CCC5C4(CCC6C5(CCC(C6(C)C)O)C)C)C)C | Yes | - 7.4 |
| 296 | oleanonic acid | 12313704 | CC1(CCC2(CCC3(C(=CCC4C3(CCC5C4(CCC(=O)C5(C)C)C)C)C2C1)C)C(=O)O)C | Yes | - 7.5 |
| 297 | Ginsenoside Rg3 | 12901617 | CC(=CCCC(C)(C1CCC2(C1C(CC3C2(CCC4C3(CCC(C4(C)C)OC5C(C(C(C(O5)CO)O)O)OC6C(C(C(C(O6)CO)O)O)O)C)C)O)C)O)C | No |  |
| 298 | Isorosmanol | 13820511 | CC(C)C1=C(C(=C2C(=C1)C3C(C4C2(CCCC4(C)C)C(=O)O3)O)O)O | yes | - 7.2 |
| 299 | ursonic acid | 13893948 | CC1CCC2(CCC3(C(=CCC4C3(CCC5C4(CCC(=O)C5(C)C)C)C)C2C1C)C)C(=O)O | Yes | - 8.3 |
| 300 | Hispidol B | 13967183 | CC(CC(C(C(C)(C)O)O)O)C1CCC2(C1(CCC3C2=CCC4C3(CCC(C4(C)C)O)C)C)C | yes | - 7.8 |
| 301 | 25-acetylcucurbitacin F | 14165733 | CC(=O)OC(C)(C)C=CC(=O)C(C)(C1C(CC2(C1(CC(=O)C3(C2CC=C4C3CC(C(C4(C)C)O)O)C)C)C)O)O | Yes | - 7.2 |
| 302 | Olean-12-en-3-alpha,24-diol | 14167252 | CC1(CCC2(CCC3(C(=CCC4C3(CCC5C4(CCC(C5(C)CO)O)C)C)C2C1)C)C)C | yes | - 7.7 |
| 303 | naviculol | 14313693 | CC1CCC2(C1(CCC(=CCO)C2C)C)C | Yes | - 5.8 |
| 304 | 3-beta-trans-p-coumaroyloxy-2-alpha-hydroxyurs-12-en-28-oic acid | 14335960 | CC1CCC2(CCC3(C(=CCC4C3(CCC5C4(CC(C(C5(C)C)OC(=O)C=CC6=CC=C(C=C6)O)O)C)C)C2C1C)C)C(=O)O | No |  |
| 305 | Rabdoumbrosanin | 14378403 | CC1(CCCC2(C1CC(C3=CC(CCC2=O)C(=C)C3=O)O)C)C | Yes | - 7.1 |
| 306 | 16-alpha- hydroxycleroda-3,13-dien-15,16-olide | 14487070 | CC1CCC2(C(C1(C)CCC3=CC(=O)OC3O)CCC=C2C)C | Yes | - 6.9 |
| 307 | Dehydroeburicoic acid | 15250826 | CC(C)C(=C)CCC(C1CCC2(C1(CC=C3C2=CCC4C3(CCC(C4(C)C)O)C)C)C)C(=O)O | yes | - 7.1 |
| 308 | Taiwaniaquinone A | 15276281 | CC(C)C1=C(C2=C(C(=O)C1=O)C3(CCCC(C3C2C=O)(C)C)C)O | Yes | - 7 |
| 309 | Taiwaniaquinol A | 15276284 | CC(C)C1=C(C2=C(C3=C1OCO3)C4(CCCC(C4C2C=O)(C)C)C)O | Yes | - 7 |
| 310 | aglycon 14-hydroxyhypocretenolide | 15378302 | CC1=CC(=O)C2=C(CCC3CC12OC(=O)C3=C)CO | Yes | - 6.3 |
| 311 | Ailanquassin A | 15432541 | CC1C2CC(=O)OC3C24COC(C1O)(C4C(C3)(C)C5C(=CC(=O)O5)C)O | Yes | - 6.7 |
| 312 | actein | 15558324 | CC1CC2(C3C(O3)(C(O2)O)C)OC4C1C5(C(CC67CC68CCC(C(C8CCC7C5(C4)C)(C)C)OC9C(C(C(CO9)O)O)O)OC(=O)C)C | No |  |
| 313 | zizyberenalic acid | 15958448 | CC(=C)C1CCC2(C1C3CCC4C(C3(CC2)C)(CCC5C4(C(=CC5(C)C)C=O)C)C)C(=O)O | Yes | - 7.4 |
| 314 | Calenduloside G6'-O-methyl ester | 16220256 | CC1(CCC2(CCC3(C(=CCC4C3(CCC5C4(CCC(C5(C)C)OC6C(C(C(C(O6)C(=O)OC)O)OC7C(C(C(C(O7)CO)O)O)O)O)C)C)C2C1)C)C(=O)O)C | No |  |
| 315 | 6-alpha-hydroxyneopulchellin | 16401759 | CC1CC2C(C(C3(C1C(CC3O)O)C)O)C(=C)C(=O)O2 | Yes | - 6.6 |
| 316 | farinosin | 16745506 | CC12CC3C(CC1C(=C)C(=O)C=C2)C(C(=O)O3)(C)O | Yes | - 6.4 |
| 317 | Melissoidesins O | 20056142 | CC(=O)OC1CCC2(C3C(CC4CC3(CC(C2C1(C)C)OC(=O)C)C(=O)C4=C)O)C | Yes | - 6.7 |
| 318 | Melissoidesins S | 20056146 | CC(=O)OC1CC(C2(C3C(CC4CC3(CC(C2C1(C)C)OC(=O)C)C(=O)C4=C)O)C)O | Yes | - 6.7 |
| 319 | 23,24-Dihydrocucurbitacin E | 21577087 | CC(=O)OC(C)(C)CCC(=O)C(C)(C1C(CC2(C1(CC(=O)C3(C2CC=C4C3C=C(C(=O)C4(C)C)O)C)C)C)O)O | Yes | - 7.4 |
| 320 | 2-deoxycucurbitacin D | 21597452 | CC1(C(=O)CCC2C1=CCC3C2(C(=O)CC4(C3(CC(C4C(C)(C(=O)C=CC(C)(C)O)O)O)C)C)C)C | Yes | - 7.3 |
| 321 | Melianin C | 21600034 | CC(=O)OC1CC2C(C(CC(C2(C3C1(C4=CCC(C4(CC3)C)C5CC(=O)OC5)C)C)OC(=O)C)OC(=O)C6=CC=CC=C6)(C)C | No |  |
| 322 | cimiracemoside F | 21606551 | CC1C2C(CC3(C2(C(CC45C3=CCC6C4(C5)CCC(C6(C)C)OC7C(C(C(CO7)O)O)O)OC(=O)C)C)C)OC8(C1OC(C8O)(C)C)O | No |  |
| 323 | (22R,23R,24R)-12-beta-acetyloxy-16-beta,23:22,25-diepoxy-23,24-dihydroxy-9,19-cyclolanostan-3-beta-yl alpha-L-arabinopyranoside | 21606551 | CC1C2C(CC3(C2(C(CC45C3=CCC6C4(C5)CCC(C6(C)C)OC7C(C(C(CO7)O)O)O)OC(=O)C)C)C)OC8(C1OC(C8O)(C)C)O | No |  |
| 324 | cimiracemoside G | 21606552 | CC1C2C(CC3(C2(C(CC45C3=CCC6C4(C5)CCC(C6(C)C)OC7C(C(C(CO7)O)O)O)OC(=O)C)C)C)OC8(C1OC(C8O)(C)C)O | No |  |
| 325 | cimiracemoside H | 21606553 | CC1C2C(CC3(C2(C(CC45C3CCC6C4(C5)CCC(C6(C)C)OC7C(C(C(CO7)O)O)O)OC(=O)C)C)C)OC8(C1OC(C8O)(C)C)O | No |  |
| 326 | (+)-7-Oxo-13-epi-pimara-14,15-dien-18-oic acid | 21606659 | CC1(CCC2C(=C1)C(=O)CC3C2(CCCC3(C)C(=O)O)C)C=C | Yes | - 7.4 |
| 327 | Cabraleadiol | 21625899 | CC1(C2CCC3(C(C2(CCC1O)C)CCC4C3(CCC4C5(CCC(O5)C(C)(C)O)C)C)C)C | yes | - 7.5 |
| 328 | Cauloside D | 21630094 | CC1C(C(C(C(O1)OC2C(OC(C(C2O)O)OCC3C(C(C(C(O3)OC(=O)C45CCC(CC4C6=CCC7C8(CCC(C(C8CCC7(C6(CC5)C)C)(C)CO)OC9C(C(C(CO9)O)O)O)C)(C)C)O)O)O)CO)O)O)O | No |  |
| 329 | colubrinic acid | 21672700 | CC(=C)C1CCC2(C1C3CCC4C(C3(CC2)C)(CCC5C4(C(C(C5(C)C)O)C=O)C)C)C(=O)O | Yes | - 7 |
| 330 | Deoxyandrographolide | 21679042 | CC1=C(C2(CCC(C(C2CC1)(C)CO)O)C)CCC3=CCOC3=O | Yes | - 7.1 |
| 331 | Bucidarasin A | 22297737 | CC1CC(C23C(C1(C)CC=C(C)C=C)CC(C=C2C(OC3OC(=O)C)OC(=O)C)OC(=O)C(C)C)O | yes | - 7.1 |
| 332 | hexanorcucurbitacin F | 23247204 | CC(=O)C1C(CC2(C1(CC(=O)C3(C2CC=C4C3CC(C(C4(C)C)O)O)C)C)C)O | Yes | - 7.3 |
| 333 | cis-3-O-p-Hydroxycinnamoyl Ursolic Acid | 24203732 | CC1CCC2(CCC3(C(=CCC4C3(CCC5C4(CCC(C5(C)C)OC(=O)C=CC6=CC=C(C=C6)O)C)C)C2C1C)C)C(=O)O | No |  |
| 334 | trans-3-O-p-Hydroxycinnamoyl Ursolic Acid | 24203733 | CC1CCC2(CCC3(C(=CCC4C3(CCC5C4(CCC(C5(C)C)OC(=O)C=CC6=CC=C(C=C6)O)C)C)C2C1C)C)C(=O)O | No |  |
| 335 | Isodihydrocostunolide | 24893995 | CC1CCCC(=CC2C(CC1)C(=C)C(=O)O2)C | yes | - 6.5 |
| 336 | Cucurbita-5,23(E)-diene-3-beta,7-beta,25-triol | 25016665 | CC(CC=CC(C)(C)O)C1CCC2(C1(CCC3(C2C(C=C4C3CCC(C4(C)C)O)O)C)C)C | yes | - 7.8 |
| 337 | acetylursolic acid | 25032406 | CC1CCC2(CCC3(C(=CCC4C3(CCC5C4(CCC(C5(C)C)OC(=O)C)C)C)C2C1C)C)C(=O)O | Yes | - 8.2 |
| 338 | Taiwaniaquinone G | 25199270 | CC(C)C1=C(C(=O)C2=C(C1=O)CC3C2(CCCC3(C)C)C)OC | Yes | - 7.1 |
| 339 | (+)-7-Oxo-13-epi-pimara-8,15-dien-18-oic acid | 38349737 | CC1(CCC2=C(C1)C(=O)CC3C2(CCCC3(C)C(=O)O)C)C=C | Yes | - 8.1 |
| 340 | Bucidarasin D | 44343868 | CC1CCC2(C(C1(C)CC=C(C)C=C)CC(=O)C=C2COC(=O)C)COC(=O)C | yes | - 6.8 |
| 341 | Cabralealactone | 44421647 | CC1(C2CCC3(C(C2(CCC1=O)C)CCC4C3(CCC4C5(CCC(=O)O5)C)C)C)C | yes | - 8.6 |
| 342 | Cabraleahydroxylactone | 44421648 | CC1(C2CCC3(C(C2(CCC1O)C)CCC4C3(CCC4C5(CCC(=O)O5)C)C)C)C | yes | - 8.4 |
| 343 | Ent-7-alpha-Hydroxykaur-16-en-15-one | 44445557 | CC1(CCCC2(C1CC(C34C2CCC(C3)C(=C)C4=O)O)C)C | yes | - 7.5 |
| 344 | Ent-7-alpha,14-beta-dihydroxykaur-16-en-15-one | 44445571 | CC1(CCCC2(C1CC(C34C2CCC(C3O)C(=C)C4=O)O)C)C | yes | - 7.6 |
| 345 | Xerophilusin B | 44445767 | CC1(CCCC23C1C(C4(C56C2CCC(C5OC3O4)C(=C)C6=O)O)O)C | Yes | - 7 |
| 346 | Macrocalin B | 44445771 | CC1(CCCC23C1C(C4(C56C2C(CC(C5OC3O4)C(=C)C6=O)O)O)O)C | yes | - 7 |
| 347 | Xerophilusin A | 44445773 | CC(=O)OC1CC2C3C4(C1C56CCCC(C5C(C4(OC6O3)O)O)(C)C)C(=O)C2=C | Yes | - 6.8 |
| 348 | xerophilusin F | 44445779 | CC(=O)OC1CC2C(C3(C1C45CCCC(C4C(=O)C3(C5O)O)(C)C)C(=O)C2=C)O | yes | - 6.7 |
| 349 | Balsaminoside A | 44555454 | CC(CC=CC(C)(C)OC)C1CCC2(C1(CCC3(C2C(C=C4C3CCC(C4(C)C)O)OC5C(C(C(C(O5)CO)O)O)O)C)C)C | yes | - 8.4 |
| 350 | Argentinic acid A methyl ester | 44566303 | CCC(C)C(C(=O)OC1CC(C(C2C1(C3=CCC(C3(CC2)C)C4CC(OC4)C=C(C)C)C)(C)CCC(=O)OC)C(C)(C)O)O | No |  |
| 351 | Argentinic acid B methyl ester | 44566304 | CC(C)C(C(=O)OC1CC(C(C2C1(C3=CCC(C3(CC2)C)C4CC(OC4)C=C(C)C)C)(C)CCC(=O)OC)C(C)(C)O)O | No |  |
| 352 | Argentinic acid C methyl ester | 44566306 | CC=C(C)C(C(=O)OC1CC(C(C2C1(C3=CCC(C3(CC2)C)C4CC(OC4)C=C(C)C)C)(C)CCC(=O)OC)C(C)(C)O)O | No |  |
| 353 | Argentinic acid E methyl ester | 44566307 | CC=C(C)C(C(=O)OC1CC(C(C2C1(C3=CCC(C3(CC2OC(=O)C(C(C)C)O)C)C4CC(OC4)C=C(C)C)C)(C)CCC(=O)OC)C(C)(C)O)O | Yes | - 7 |
| 354 | Argentinic acid F methyl ester | 44566308 | CCC(C)C(C(=O)OC1CC2(C(CC=C2C3(C1C(C(CC3OC(=O)C(C(C)C)O)C(C)(C)O)(C)CCC(=O)OC)C)C4CC(OC4)C=C(C)C)C)O | Yes | - 6.6 |
| 355 | Argentinic acid G methyl ester | 44566309 | CCC(C)C(C(=O)OC1CC(C(C2C1(C3=CCC(C3(CC2OC(=O)C(C(C)CC)O)C)C4CC(OC4)C=C(C)C)C)(C)CCC(=O)OC)C(C)(C)O)O | Yes | - 6.4 |
| 356 | Argentinic acid H methyl ester | 44566310 | CC=C(C)C(C(=O)OC1CC(C(C2C1(C3=CCC(C3(CC2OC(=O)C(C(=CC)C)O)C)C4CC(OC4)C=C(C)C)C)(C)CCC(=O)OC)C(C)(C)O)O | Yes | - 7.4 |
| 357 | Calenduloside F6'-O-n butyl ester | 44566502 | CCCCOC(=O)C1C(C(C(C(O1)OC2CCC3(C(C2(C)C)CCC4(C3CC=C5C4(CCC6(C5CC(CC6)(C)C)C(=O)OC7C(C(C(C(O7)CO)O)O)O)C)C)C)O)O)O | No |  |
| 358 | Meliavolkinin | 44566525 | CC(=O)OC1CC(C2(C3CCC4(C(CC=C4C3(C(C5C2C1(CO5)C)O)C)C6=COC=C6)C)C)OC(=O)C7=CC=CC=C7 | yes | - 7.6 |
| 359 | 1,3-diacetylvilasinin | 44566526 | CC(=O)OC1CC(C2(C3CCC4(C(CC=C4C3(C(C5C2C1(CO5)C)O)C)C6=COC=C6)C)C)OC(=O)C | Yes | - 7.9 |
| 360 | Melianin B | 44566528 | CC(=O)OC1CC2C(C(CC(C2(C3C1(C4=CCC(C4(CC3)C)C5CC(C(C(OC5)(C)C)O)O)C)C)OC(=O)C)OC(=O)C6=CC=CC=C6)(C)C | No |  |
| 361 | 1-beta,2-alpha-epoxytagitinin C | 44566960 | CC1=CC2C(C(CC(C3C(C1=O)O3)(C)O)OC(=O)C(C)C)C(=C)C(=O)O2 | Yes | - 6.9 |
| 362 | 9,13-diacetyltaxumairol W | 44567148 | CC1=C2C(C(C3(C(CC4C(C3C(C2(CC1OC(=O)C)C(C)(C)O)OC(=O)C)(CO4)OC(=O)C)OC(=O)C)C)OC(=O)C)OC(=O)C | No |  |
| 363 | Guanepolide | 44575525 | CC(=O)OC1CC(C2=CC(=O)OC3C2(C1C4(C(C3)C(C=CC4=O)(C)O)C)C)(C)C5C(CC(=O)O5)CO | yes | - 7.9 |
| 364 | Polyandrol | 44575526 | CC1C2C(C(=O)OC3C24COC(C1O)(C4C(C3)(C)C5C(=CC(=O)O5)C)O)O | yes | - 6.8 |
| 365 | Uncarinic acid C | 44583694 | CC1CCC2(CCC3(C(=CCC4C3(CCC5C4(CCC(C5(C)C)O)C)C)C2C1C)CC(=O)C=CC6=CC(=C(C=C6)O)OC)C(=O)O | No |  |
| 366 | Uncarinic acid D | 44583695 | CC1CCC2(CCC3(C(=CCC4C3(CCC5C4(CCC(C5(C)C)O)C)C)C2C1C)CC(=O)C=CC6=CC(=C(C=C6)O)OC)C(=O)O | No |  |
| 367 | Uncarinic acid E | 44583696 | CC1(CCC2(CCC3(C(=CCC4C3(CCC5C4(CCC(C5(C)C)O)C)C)C2C1)COC(=O)C=CC6=CC=C(C=C6)O)C(=O)O)C | No |  |
| 368 | Xindongnin B | 44584350 | CC(=O)OC1C2C(C(CCC2(C3C(CC4CC3(C1O)C(=O)C4=C)O)C)O)(C)C | Yes | - 7 |
| 369 | Buddledin B | 44593338 | CC1=CCCC(=C)C2CC(C2C(C1=O)O)(C)C | yes | - 6.3 |
| 370 | Methylmitrekaurenate | 44606073 | CC1(CCCC(C1C(=O)O)(C)C(=O)OC)C2CCC3CC2(CC3=C)C(=O)O | Yes | - 6.6 |
| 371 | Balsaminapentaol A | 44607275 | CC(CC(C(C(=C)C)O)O)C1CCC2(C1(CCC3(C2C(C=C4C3CCC(C4(C)CO)O)O)C)C)C | yes | - 6.7 |
| 372 | Balsaminol A | 44607276 | CC(CC(C=C(C)C)O)C1CCC2(C1(CCC3(C2C(C=C4C3CCC(C4(C)CO)O)O)C)C)C | yes | - 7.2 |
| 373 | Balsaminol B | 44607277 | CC(CC(C=C(C)C)O)C1CCC2(C1(CCC3(C2C(C=C4C3CCC(C4(C)CO)O)OC)C)C)C | yes | - 6.8 |
| 374 | Cucurbalsaminol A | 44607278 | CC(CC=CC(C)(C)O)C1CCC2(C1(C(CC3(C2C(C=C4C3CCC(C4(C)C)O)O)C)O)C)C | yes | - 7.7 |
| 375 | Brunneogaleatoside | 45027872 | CC1(CCC2=C(C(OC(C21)OC3C(C(C(C(O3)COC(=O)C=CC4=CC=C(C=C4)O)O)O)O)OC)C(=O)OC)O | No |  |
| 376 | Hexanorcucurbitacin D | 45272227 | CC(=O)C1C(CC2(C1(CC(=O)C3(C2CC=C4C3CC(C(=O)C4(C)C)O)C)C)C)O | yes | - 7.3 |
| 377 | Toosendanin | 45358155 | CC(=O)OC1CC(C23COC(C1(C2CC(C4(C3C(=O)C(C5(C46C(O6)CC5C7=COC=C7)C)OC(=O)C)C)O)C)O)O | No |  |
| 378 | Altaicalarins A | 46186370 | CC=C(C)C(=O)OC(CC=C(C)C)C(=C)C1=CC(=C(C(=C1OC(=O)C)O)C)O | yes | - 6.3 |
| 379 | Altaicalarins B | 46186371 | CC=C(C)C(=O)OC(CC=C(C)C)C(=C)C1=C(C(=C(C=C1)C)O)O | yes | - 7.1 |
| 380 | Altaicalarins C | 46186620 | CC=C(C)C(=O)OC(CC=C(C)C)C(=C)C1CC2C(O2)(C(=O)C1OC(=O)C)C | yes | - 6.3 |
| 381 | Altaicalarins D | 46186621 | CC=C(C)C(=O)OC1C(C(C(=O)C2(C1O2)C)OC(=O)C)C(=C)C(CC3C(O3)(C)C)OC(=O)C(=CC)C | yes | - 6.1 |
| 382 | Englerin A | 46242512 | CC1CCC2C1C(C3(CC(C2(O3)C)OC(=O)CO)C(C)C)OC(=O)C=CC4=CC=CC=C4 | Yes | - 7.7 |
| 383 | longilactone | 46878941 | CC1C(C(C2C3(C1C(=O)OC3C(C4C2(C(C(=O)C=C4C)O)C)O)C)O)O | yes | - 6.9 |
| 384 | Taiwaniaquinol F | 46880979 | CC(C)C1=C(C2=C(C(=C1OC)O)C3(CCC(=O)C(C3C2=O)(C)C)C)O | Yes | - 6.8 |
| 385 | Taiwaniaquinone E | 46881290 | CC(C)C1=C(C2=C(C(=O)C1=O)C3(CCCC(C3C2C(=O)OC)(C)C)C)O | Yes | - 6.3 |
| 386 | Karavilagenin E | 46882748 | CC(CC(C=C(C)C)O)C1CCC2(C1(CCC34C2C=CC5(C3CCC(C5(C)C)O)OC4)C)C | yes | - 7.3 |
| 387 | Balsaminols E | 46912852 | CC(CC(C=C(C)C)O)C1CCC2(C1(CCC3(C2C(=O)C=C4C3CCC(C4(C)C)O)C)C)C | yes | - 8.4 |
| 388 | Balsaminols C | 52947022 | CC(CC(=O)C=C(C)C)C1CCC2(C1(CCC3(C2C(=O)C=C4C3CCC(C4(C)CO)O)C)C)C | yes | - 8.2 |
| 389 | Balsaminols D | 52947048 | CC(CC(=O)C)C1CCC2(C1(CCC3(C2C(=O)C=C4C3CCC(C4(C)CO)O)C)C)C | yes | - 7.9 |
| 390 | Methyl zhankuic acid A | 53319974 | CC1C2CC(=O)C3=C(C2(CCC1=O)C)C(=O)CC4(C3CCC4C(C)CCC(=C)C(C)C(=O)OC)C | yes | - 8.6 |
| 391 | Resiniferol 20-(4-hydroxy-3-methoxyphenylacetate) 9,13,14-ortho-phenylacetate | 53776296 | CC1CC2(C3C4C1(C5C=C(C(=O)C5(CC(=C4)COC(=O)CC6=CC(=C(C=C6)O)OC)O)C)OC(O3)(O2)CC7=CC=CC=C7)C(=C)C | yes | - 8.4 |
| 392 | Cedronolactones C | 10714787 | CC1C2C(C(=O)OC3C24COC(C1O)(C4C(C3)(C)C5C(=CC(=O)O5)C)O)O | yes | - 6.4 |
| 393 | 25-O-methoxycimigenol 3-O-alpha-L-arabinopyranoside | 11856241 | CC1CC2C(OC3(C1C4(CCC56CC57CCC(C(C7CCC6C4(C3O)C)(C)C)OC8C(C(C(CO8)O)O)O)C)O2)C(C)(C)OC | Yes | - 6.8 |
| 394 | 12-beta,21-dihydroxycimigenol 3-O-alpha-L-arabinopyranoside | 11856500 | CC1(C2CCC3C4(C(C56C(C4(C(CC37C2(C7)CCC1OC8C(C(C(CO8)O)O)O)O)C)C(CC(O5)C(O6)C(C)(C)O)CO)O)C)C | No |  |
| 395 | Saikosaponin a | 167928 | CC1C(C(C(C(O1)OC2CCC3(C(C2(C)CO)CCC4(C3C=CC56C4(CC(C7(C5CC(CC7)(C)C)CO6)O)C)C)C)O)OC8C(C(C(C(O8)CO)O)O)O)O | Yes | - 6.4 |
| 396 | Weisiensin A | 377386 | CC(=O)OC1CC2CC3(C1C4(C(CC(C(C4C(C3OC(=O)C)O)(C)C)O)OC(=O)C)C)C(=O)C2=C | Yes | - 6.6 |
| 397 | Saikosaponin b2 | 45358150 | CC1C(C(C(C(O1)OC2CCC3(C(C2(C)CO)CCC4(C3C=CC5=C6CC(CCC6(C(CC54C)O)CO)(C)C)C)C)O)OC7C(C(C(C(O7)CO)O)O)O)O | No |  |
| 398 | 3-beta-Hydroxy-27-p-(E)-coumaroyloxyurs-12-en-28-oic acid | 6306917 | CC1CCC2(CCC3(C(=CCC4C3(CCC5C4(CCC(C5(C)C)O)C)C)C2C1C)COC(=O)C=CC6=CC=C(C=C6)O)C(=O)O | No |  |

**Table S2** Molecular docking results and toxicity profiles of terpenoid compound having lower binding energy that standard inhibitor, Nutlin-3a (binding energy: - 8.8 kcal/mol)

| **Sl no.** | **Compound Name** | **PubChem ID** | **SMILES** | **Binding energy (kcal/mol)** | **Carcinogenicity** | **Mutagenicity** |
| --- | --- | --- | --- | --- | --- | --- |
| 1 | Lupeol | 259846 | CC(=C)C1CCC2(C1C3CCC4C5(CCC(C(C5CCC4(C3(CC2)C)C)(C)C)O)C)C | - 8.8 | Inactive | Inactive |
| 2 | Remangilones C | 397856 | CC1=C(C(=O)CC2(C1CCC3(C2CC=C4C3(C(C(=O)C5C4CC(CC5)(C)C)O)C)C)C)O | - 8.7 | **Active** | Inactive |
| 3 | Epi-betulinic acid | 485711 | CC(=C)C1CCC2(C1C3CCC4C5(CCC(C(C5CCC4(C3(CC2)C)C)(C)C)O)C)C(=O)O | - 8.7 | **Active** | Inactive |
| 4 | Betulinic acid | 64971 | CC(=C)C1CCC2(C1C3CCC4C5(CCC(C(C5CCC4(C3(CC2)C)C)(C)C)O)C)C(=O)O | - 8.6 | **Active** | Inactive |
| 5 | Alpha-amyrin | 73170 | CC1CCC2(CCC3(C(=CCC4C3(CCC5C4(CCC(C5(C)C)O)C)C)C2C1C)C)C | - 8.6 | Inactive | Inactive |
| 6 | 27-deoxyactein | 6537501 | CC1CC2(C3C(O3)(CO2)C)OC4C1C5(CCC67CC68CCC(C(C8CCC7C5(C4)C)(C)C)OC9C(C(C(CO9)O)O)O)C | - 8.6 | Inactive | Inactive |
| 7 | Cabralealactone | 44421647 | CC1(C2CCC3(C(C2(CCC1=O)C)CCC4C3(CCC4C5(CCC(=O)O5)C)C)C)C | - 8.6 | Inactive | Inactive |
| 8 | Methyl zhankuic acid A | 53319974 | CC1C2CC(=O)C3=C(C2(CCC1=O)C)C(=O)CC4(C3CCC4C(C)CCC(=C)C(C)C(=O)OC)C | - 8.6 | **Active** | Inactive |
| 9 | Celastrol | 122724 | CC1=C(C(=O)C=C2C1=CC=C3C2(CCC4(C3(CCC5(C4CC(CC5)(C)C(=O)O)C)C)C)C)O | - 8.5 | Inactive | Inactive |
| 10 | Olean-12-en-3-beta-ol | 6708529 | CC1(CCC2(CCC3(C(=CCC4C3(CCC5C4(CCC(C5(C)C)O)C)C)C2C1)C)C)C | - 8.5 | Inactive | Inactive |
| 11 | Cabraleahydroxylactone | 44421648 | CC1(C2CCC3(C(C2(CCC1O)C)CCC4C3(CCC4C5(CCC(=O)O5)C)C)C)C | - 8.4 | Inactive | Inactive |
| 12 | Alphitolic acid | 470606 | CC(=C)C1CCC2(C1C3=CCC4C(C3(CC2)C)(CCC5C4(CC(C(C5(C)C)O)O)C)C)C(=O)O | - 8.4 | **Active** | Inactive |
| 13 | Sulphurenic acid | 5321559 | CC(C)C(=C)CCC(C1CC(C2(C1(CCC3=C2CCC4C3(CCC(C4(C)C)O)C)C)C)O)C(=O)O | - 8.4 | Inactive | Inactive |
| 14 | Balsaminoside A | 44555454 | CC(CC=CC(C)(C)OC)C1CCC2(C1(CCC3(C2C(C=C4C3CCC(C4(C)C)O)OC5C(C(C(C(O5)CO)O)O)O)C)C)C | - 8.4 | Inactive | Inactive |
| 15 | Balsaminols E | 46912852 | CC(CC(C=C(C)C)O)C1CCC2(C1(CCC3(C2C(=O)C=C4C3CCC(C4(C)C)O)C)C)C | - 8.4 | **Active** | Inactive |
| 16 | Resiniferol 20-(4-hydroxy-3-methoxyphenylacetate) 9,13,14-ortho-phenylacetate | 53776296 | CC1CC2(C3C4C1(C5C=C(C(=O)C5(CC(=C4)COC(=O)CC6=CC(=C(C=C6)O)OC)O)C)OC(O3)(O2)CC7=CC=CC=C7)C(=C)C | - 8.4 | Inactive | Inactive |
| 17 | Canophyllic acid | 596679 | CC1C(CCC2C1(CCC3C2(CCC4(C3(CCC5(C4CC(CC5)(C)C)C(=O)O)C)C)C)C)O | - 8.4 | Inactive | Inactive |
| 18 | Isoiguesterol | 10477355 | CC1=C(C(=O)C=C2C1=CC=C3C2(CCC4(C3(CCC5(C4CC(CC5)CO)C)C)C)C)O | - 8.3 | Inactive | Inactive |
| 19 | Ursonic acid | 13893948 | CC1CCC2(CCC3(C(=CCC4C3(CCC5C4(CCC(=O)C5(C)C)C)C)C2C1C)C)C(=O)O | - 8.3 | **Active** | Inactive |

**S3** List of clusters obtained from by clustering 250 ns MD simulation trajectory data of unbound MDM2 protein

| **Cluster No** | **RMSD** | **Number of Structure** | **Percentage of total structure population** |
| --- | --- | --- | --- |
| Cluster-1 | 0.118 | 20703 | 82.809% |
| Cluster-2 | 0.104 | 2335 | 9.340% |
| Cluster-3 | 0.122 | 1759 | 7.036% |
| Cluster-4 | 0.126 | 102 | 0.408% |
| Cluster-5 | 0.143 | 44 | 0.176% |
| Cluster-6 | 0.126 | 16 | 0.064% |
| Cluster-7 | 0.143 | 11 | 0.044% |
| Cluster-8 | 0.138 | 10 | 0.039% |
| Cluster-9 | 0.073 | 6 | 0.023% |
| Cluster-10 | 0.073 | 5 | 0.019% |
| Cluster-11 | 0.183 | 3 | 0.011% |
| Cluster-12 | 0.124 | 3 | 0.011% |
| Cluster-13 | 0.127 | 2 | 0.008% |
| Cluster-14 | - | 1 | 0.003% |
| Cluster-15 | - | 1 | 0.003% |

**Table S4** List of Protein-ligand interactions of top three selected compound with top conformations of amdm2 protein

| **Compound Name** | **Cluster** | **Binding energy (kcal/mol**) | **Interacting residues** | |
| --- | --- | --- | --- | --- |
|  |  |  | **H**- **Bonding** | **Hydrophobic**  **Interaction** |
| Olean-12-en-3-beta-ol | Cluster-1 | - 7.9 | - | Met-62, Val-93, Leu-54, Ile-99, Ile-61, Tyr-67, Try-100 |
|  | Cluster-2 | - 8.2 | **-** | Met-62, Val-93, Leu-54, Ile-61 |
|  | Cluster-3 | - 8.3 | Tyr-100 | Met-62, Val-93, Ile-99, Leu-54, Ile-61, Tyr-67, Try-100 |
| 27-deoxyactein | Cluster-1 | - 8.3 | Gly-58 | Leu-54, Ile-99, Ile-61, Val-75, Val-93, Phe-55, Tyr-67, Val-93 |
|  | Cluster-2 | - 7.9 | Thr-49 | Lys-51, Leu-54, Val-93, Phe-55 |
|  | Cluster-3 | - 7.6 | - | Leu-54, Lys-51, Ile-99 |
| Cabralealactone | Cluster-1 | - 8.5 | Gln-72 | Leu-54, Val-93, Ile-61, Val-75 |
|  | Cluster-2 | - 7.8 | **-** | Leu-54, Ile-61, Val-75, Val-93, |
|  | Cluster-3 | - 7.5 | **-** | Leu-54, Ile-61, Val-93 |
